# BRAF Inhibition–Associated Nuclear Remodeling is Linked to Cancer-Associated Fibroblast Activation

**DOI:** 10.1158/2767-9764.CRC-25-0682

**Published:** 2026-07-16

**Authors:** Jie Wang, Bruna da Silva Soley, Yao Xiao, Lindsey G. Siegfried, Linli Zhou, Mingang Xu, Sarah Millar, Thomas Andl, Yuhang Zhang

**Affiliations:** 1 Division of Pharmaceutical Sciences, College of Pharmacy, University of Cincinnati, Cincinnati, Ohio.; 2 Black Family Stem Cell Institute, Icahn School of Medicine at Mount Sinai, New York, New York.; 3 Department of Cell, Developmental and Regenerative Biology, Icahn School of Medicine at Mount Sinai, New York, New York.; 4 Department of Oncological Sciences, Icahn School of Medicine at Mount Sinai, New York, New York.; 5 Tisch Cancer Institute, Icahn School of Medicine at Mount Sinai, New York, New York.; 6 Burnett School of Biological Sciences, University of Central Florida, Orlando, Florida.

## Abstract

Cancer-associated fibroblasts (CAFs) display remarkable plasticity, enabling them to adapt to therapeutic and mechanical stress within the tumor microenvironment. In this study, we identify a shared mechanotransduction pathway by which BRAF inhibition and matrix stiffness converge on ROCK-dependent cytoskeletal remodeling, nuclear deformation, and β-catenin nuclear accumulation in CAFs. Mechanistically, BRAF inhibitors (BRAFi) accelerate RAS-dependent RAF homodimerization and heterodimerization and promote ERK signaling, accompanied by GSK-3β inactivation and activation of the ROCK pathway. ROCK activation induces actin stress fiber assembly and nuclear deformation. Stiff substrates recapitulate BRAFi-induced actin remodeling and nuclear deformation in CAFs. In both contexts, nuclear remodeling is associated with β-catenin nuclear accumulation and CAF activation. Functionally, constitutive β-catenin activation in mouse fibroblasts enhanced CAF-like features *in vitro* and promoted melanoma growth and matrix remodeling *in vivo*. Pharmacologic ROCK inhibition blocked both BRAFi- and stiffness-induced nuclear remodeling and β-catenin accumulation, identifying the ROCK–cytoskeleton–nucleus axis as a mediator of CAF responses to therapeutic and mechanical cues. Collectively, these findings reveal a mechanically tuned signaling mechanism that contributes to CAF activation, supporting ROCK inhibition as a potential strategy to limit tumor-promoting stromal adaptation during targeted therapy.

**Significance:**

This study shows that CAFs respond to BRAF inhibition and mechanical cues via a shared ROCK–cytoskeleton–nucleus pathway. ROCK-dependent nuclear remodeling is associated with β-catenin accumulation and CAF activation, whereas ROCK inhibition disrupts this response, highlighting a mechanotransduction pathway that may contribute to stromal adaptation during targeted therapy.

## Introduction

Cancer-associated fibroblasts (CAFs) are a functionally versatile stromal population in the tumor microenvironment (TME), contributing to tumor progression, metastatic dissemination, and therapy resistance (1). Arising from diverse cellular origins, including resident fibroblasts, mesenchymal stem cells, or other progenitor cells, CAFs exhibit a reprogrammed phenotype marked by the expression of biomarkers such as α–smooth muscle actin (α-SMA), fibroblast activation protein, and platelet-derived growth factor receptor-β (2–4). Functionally, CAFs promote tumor progression by remodeling the extracellular matrix (ECM), secreting protumorigenic cytokines and growth factors, and engaging in dynamic cross-talk with malignant cells and other stromal cells, thereby shaping the biochemical and mechanical landscape of the TME (4).

Within the TME, CAFs encounter and integrate a wide spectrum of biophysical and biochemical signals, including therapeutic agents, ECM stiffness, and metabolic byproducts, which modulate their activation state, phenotype, and function. Mechanical stress resulting from matrix stiffening and ECM remodeling activates mechanotransduction pathways in CAFs, including integrin signaling and YAP/TAZ nuclear translocation, which increase cytoskeletal tension and further reinforce CAF activation (5, 6). In this activated state, CAFs secrete prosurvival and immunosuppressive factors and contribute to a fibrotic ECM that impedes drug delivery and fosters therapeutic resistance (4). Equally important, therapeutic agents, ranging from cytotoxic drugs to targeted therapies, are emerging as active participants in remodeling the TME and exerting selective pressure on both tumor and stromal cells (7). However, how pharmacologic signals and mechanical cues converge to regulate CAF activation remains poorly understood.

One of the most transformative advances in the treatment of metastatic melanoma has been the development of BRAF inhibitors (BRAFi) targeting the V600E mutation in the BRAF kinase domain (8). This mutation allows BRAF to bypass autoinhibition and constitutively activate the downstream MAPK/ERK pathway, driving tumor growth (9–12). BRAFi, such as vemurafenib and dabrafenib, effectively block this aberrant signaling; however, therapeutic resistance frequently emerges, limiting long-term patient benefit (13, 14). Although BRAFi were designed to selectively target mutant cancer cells, their effects on stromal cells, including CAFs that carry wild-type (WT) BRAF, are increasingly recognized as critical factors influencing therapeutic response and resistance (15, 16). Growing evidence has revealed paradoxical activation of the MAPK/ERK pathway in WT BRAF- or RAS-mutant cells via BRAFi-induced RAF dimerization (17–19). Despite these findings, the mechanisms by which BRAFi modulate CAFs and interact with mechanical signaling pathways remain unresolved.

In this study, we sought to delineate how pharmacologic and mechanical signals regulate adaptive responses in CAFs during melanoma therapy. We show that BRAFi and matrix stiffness regulate CAF responses by activating the ROCK signaling pathway, which promotes actin stress fiber assembly and nuclear deformation. This cytoskeletal–to–nuclear mechanical relay is associated with β-catenin nuclear accumulation, implicating β-catenin as a common downstream effector of pharmacologic and mechanical inputs. Together, these results identify actin-mediated nuclear remodeling as a ROCK-dependent mechanism through which CAFs integrate pharmacologic and mechanical cues in the melanoma microenvironment.

## Materials and Methods

### Transgenic mouse strains

*Col1α2-CreER* mice [*B6.Cg-Tg(Col1α2-cre/ERT,-ALPP)7Cpd/2J*; RRID: IMSR_JAX:029567] were kindly provided by Dr. de Crombrugghe (20). The *tetO-ΔN-β-catenin* transgenic mouse strain was generated in S. Millar laboratory and was reported in our previous studies (21, 22). The *Rosa-rtTA* mouse strain [*B6.Cg-Gt(ROSA)26Sor*^*tm1(rtTA,EGFP)Nagy*^*/J*; RRID: IMSR_JAX:005670] was obtained from The Jackson Laboratory. Both mouse strains were backcrossed to the C57BL/6 (B6) genetic background. To generate the triple *Col1α2-CreER*; *Rosa-rtTA*; *tetO-ΔN-β-catenin* mouse strain, *Col1α2-CreER*, *Rosa-rtTA*, and *tetO-ΔN-β-catenin* mice were crossed for several generations. Mice were genotyped by polymerase chain reaction (PCR) of genomic DNA extracted from tail biopsies. The presence of the Cre transgene, *Col1α2-CreER*, was identified by PCR using the forward primer, 5′ CGG​TCT​GGC​AGT​AAA​AAC​TAT 3′, and the reverse primer, 5′ CAG​GGT​GTT​ATA​AGC​AAT​CCC 3′. The *Rosa-rtTA* allele was genotyped using the forward primer, 5′ AAG​ACC​GCG​AAG​AGT​TTG​TC 3′, and the reverse primer, 5′ AAA​GTC​GCT​CTG​AGT​TGT​TAT 3′. The *ΔN-β-catenin* transgene was genotyped using the forward primer, 5′ CCT​TGT​ATC​ACC​ATG​GAC​CCT​CAT 3′, and the reverse primer: 5′ TAG​TGG​GAT​GAG​CAG​CGT​CAA​ACT 3′. Standard PCR protocols were used. Male and female animals were used in the study, and no differences were observed between sexes. All mice were housed in the Laboratory Animal Services Facility of the University of Cincinnati under an artificial 14/10-hour light–dark cycle and allowed free access to food and water.

### Cell lines

Human melanoma cell lines A375 (cat. #CRL-1619, RRID: CVCL_0132) and SK-MEL-24 (cat. #HTB-71, RRID: CVCL_0599) were purchased from the American Type Culture Collection (ATCC). Two human CAF cell lines, 224350P1 and DT01027P1, isolated from surgically excised human melanoma tissues, were obtained from Asterand Bioscience, designated as M50 and M27, respectively, and previously authenticated (7). Human dermal fibroblasts, referred to as normal fibroblasts (NFs) in the experiments, were provided by Dr. Dorothy Supp of the Department of Surgery at the University of Cincinnati. Murine *Braf*^*V600E*^; *Pten*^*lox5/lox5*^ D4M melanoma cells were purchased from Kerafast (23). Melanoma cell lines were authenticated by the ATCC and routinely validated in our laboratory based on the expression of melanoma proteoglycan antigens and surface molecules, including GP100 (24), MITF, melan-A, and tyrosinase (25). We authenticated CAFs and normal human dermal fibroblasts by evaluating their unique morphologic characteristics and expression of specific skin fibroblast markers, including TE7 (26), vimentin, and α-SMA (27). All cell lines were passaged fewer than 20 times and maintained in Dulbecco’s modified Eagle medium (DMEM) supplemented with 10% (v/v) fetal bovine serum (FBS) and 1% penicillin–streptomycin solution (Corning, 30-002-CL) in a humidified incubator at 37°C with 5% CO_2_. All cell lines were routinely tested for *Mycoplasma* contamination using a PCR-based Universal Mycoplasma Detection Kit (ATCC, 30-1012K). Cells were confirmed to be *Mycoplasma*-free prior to use in experiments. Cell culture reagents were purchased from Corning Life Sciences unless otherwise stated. Experimental procedures involving biosafety hazards were performed under the University of Cincinnati Institutional Biosafety Committee protocol 16-08-17-01.

### Mouse primary fibroblast isolation

The sex of neonatal *Col1α2-CreER*, *Rosa-rtTA*, and *TetO-ΔN-β-catenin* mice was first determined by genotyping the *SRY* gene using PCR with the forward primer, 5′ TTG​TCT​AGA​GAG​CAT​GGA​GGG​CCA​TGT​CAA 3′, and the reverse primer, 5′ CCA​CTC​CTC​TGT​GAC​ACT​TTA​GCC​CTC​CGA 3′. To isolate dermal fibroblasts, the dorsal skin of sex-matched 2- to 3-day-old littermates was collected, and subcutaneous fat and other tissues were removed from the dermal side. The skin was floated with the epidermis facing up in a 0.25% trypsin solution at 37°C for 1 hour to separate the dermis and epidermis. The dermis was minced into small pieces of approximately 1 mm^2^ using sterile scissors. The dermal pieces were then incubated in 5 mL of 2.5 mg/mL collagenase IV solution for 30 minutes at 37°C with pipetting every 10 minutes. After 30 minutes, 5 mL of DMEM was added to stop the digestion. The dermal cell suspension was filtered through 70- and 40-μm strainers to remove cell clumps and undissociated tissue. Fibroblasts were collected by centrifugation at 1,500 rpm for 5 minutes and resuspended in 10 mL of DMEM for culture in a 10-cm dish. Fibroblast genotypes were determined by PCR as described for transgenic mouse strains and validated by immunostaining for the expression of α-SMA, vimentin, S100A4, keratin 14 (negative, keratinocyte marker), and TRP1 (negative, melanocyte marker). Four pairs of ΔN-β-catenin–overexpressing mouse fibroblasts and WT fibroblasts were isolated and frozen in liquid nitrogen at passages 3 to 5 for *in vitro* and *in vivo* studies.

### Induction of ΔN-β-catenin expression in cultured mouse fibroblasts

To activate CreER- and rtTA-dependent ΔN-β-catenin expression in isolated mouse fibroblasts, 3 × 10^5^ cells were seeded in a 10-cm dish and treated with 4-hydroxytamoxifen (Sigma-Aldrich, H6278) at a final concentration of 0.2 μg/mL and doxycycline (Dox; Sigma-Aldrich, 33429) at 1.25 μg/mL for 72 hours. Following induction, cells were washed with PBS and harvested for subsequent assays.

### Cell proliferation assay

Dox-treated mouse fibroblasts (1 × 10^5^) of the genotypes *Col1α2-CreER*; *Rosa-rtTA*; *TetO-ΔN-β-catenin* or *Col1α2-CreER*; *Rosa-rtTA* were seeded and cultured in a 6-cm dish. On days 3, 5, and 7, the cells were collected, and the number of cells was counted using a hemocytometer. At least three repeats were included for each indicated cell line. Cell counting was performed in at least three independent experiments for statistical analysis.

### Collagen gel contraction assay

Dox-treated 5 × 10^4^ mouse fibroblasts of the genotypes *Col1α2-CreER*; *Rosa-rtTA*; *TetO-ΔN-β-catenin* and *Col1α2-CreER*; *Rosa-rtTA* were suspended in 500 μL of 1 mg/mL rat tail collagen type I (Advanced BioMatrix, cat. #5153) in DMEM and seeded in one well of a 24-well tissue culture plate. After 30 minutes of incubation in a humidified incubator at 37°C, 1 mL of fresh medium was added to the top of the gel for incubation. After 72 hours of incubation, the gel was detached from the wall of each well and allowed to contract as indicated. Images of the gels were taken using a Nikon digital camera every 24 hours, and ImageJ software (NIH) was used to measure the area of the gel. Gel contraction was calculated as the percentage reduction in the gel area from 0 to 72 hours: gel contraction (%) = [(area at 0 hours − area at 72 hours)/area at 0 hours] × 100.

### Confocal reflection microscopy

Dox-treated mouse fibroblasts (5 × 10^4^) of the genotypes *Col1α2-CreER*; *Rosa-rtTA*; *TetO-ΔN-β-catenin* or *Col1α2-CreER*; *Rosa-rtTA* were embedded in collagen gels in the same way as described for the collagen gel contraction assay. After 72 hours of incubation, the gels were collected, and collagen fiber distribution and organization were directly examined using a Zeiss LSM 710 confocal microscope at 40× magnification. Images were acquired at a depth of at least 100 μm below the top surface of the gel to minimize the edge effects. Images of at least 10 areas of each gel were randomly captured for three-dimensional (3D) reconstruction using ImageJ. Fiber connectivity and mean spacing were calculated using ImageJ with the BoneJ plugin (28).

### Mouse melanoma induction

As shown in Fig. 3A, two groups of melanomas were generated by intradermal injection of a mixture of 1 × 10^5^ D4M melanoma cells and 1 × 10^5^ uninduced mouse fibroblasts of the genotype *Col1α2-CreER*; *Rosa-rtTA*; *TetO-ΔN-β-catenin* or *Col1α2-CreER*; *Rosa-rtTA* into the flanks of 5-week-old B6 mice. For each group, a minimum of three male and three female mice were used as recipient mice. The mice were monitored daily after cell injection for tumor formation. After the tumors reached a volume of approximately 62.5 mm^3^ (designated as day zero), to induce β-catenin overexpression in *Col1α2-CreER*; *Rosa-rtTA*; *TetO-ΔN-β-catenin* fibroblasts, all mice were fed a Dox diet (Bio-Serv, F-4096) and underwent intraperitoneal injection of 10 mg/mL tamoxifen (Sigma-Aldrich, T5648) in corn oil at 1 mg/g body weight for seven consecutive days. Afterward, the tumor size was measured using a caliper every other day for 18 days. Tumor volume was calculated using the following formula: [(width)^2^ × (length)]/2. The mice were euthanized around day 18 when the tumor size exceeded 15% of the body size, and the tumors were harvested for analysis. The number of mice used in each study was calculated using a power analysis. Animals were allocated to experimental groups based on genotype and experimental design. Student *t* test was used to determine the significance of differences in tumor size and weight between groups.

### Histology, immunofluorescence staining, and immunohistochemistry

The collected melanoma tissues were fixed in 10% formalin overnight at 4°C and embedded in paraffin for sectioning. Five-μm-thick paraffin-embedded tumor tissue sections were prepared for hematoxylin and eosin (H&E) staining and immunostaining as described previously (7, 29). For histologic analysis, paraffin sections were stained using a standard H&E staining protocol, mounted with VectaMount permanent mounting medium (Vector Laboratories, H-1000), and analyzed using a Nikon Eclipse 80i fluorescence microscope.

For immunostaining, paraffin sections were deparaffinized, rehydrated, and unmasked in citrate buffer (pH 6.0) using the microwave heating method. After washing with PBS, the sections were blocked with 10% bovine serum albumin (BSA) and subsequently incubated with each primary antibody at 4°C overnight. The following primary antibodies were used: anti–α-SMA (Thermo Fisher Scientific, cat. #14-9760-82, RRID: AB_2572996, 1:200), anti-fibronectin (Millipore, cat. #F3648, RRID: AB_476976, 1:200), anti-Ki67 (Proteintech, cat. #27309-1-AP, RRID: AB_2756525, 1:50), and anti-cyclin D1 (Thermo Fisher Scientific, cat. #MA5-14512, RRID: AB_10985779, 1:50). After incubation with primary antibodies, the slides were washed with PBS three times, incubated with the corresponding biotin-conjugated secondary antibodies at room temperature for 1 hour, washed again three times with PBS, and then incubated with either fluorochrome-conjugated streptavidin for immunofluorescence (IF) or VECTASTAIN Elite ABC Reagents (Vector Laboratories, PK-7100) for immunohistochemistry (IHC). Nuclei were counterstained with 4′,6-diamidino-2-phenylindole (DAPI; blue) for IF and hematoxylin (blue) for IHC. Images were captured using a Nikon Eclipse 80i fluorescence microscope.

The numbers of positively stained cells (α-SMA+, Ki67+, cyclin D1+) or α-SMA− melanoma cells in each high-power field (40×) were counted using the particle analysis function of ImageJ. The number of cells per square millimeter was calculated by multiplying the number of cells counted in each field by 4.5. The fibronectin+ area in each high-power field (40×) was measured using the ImageJ software. The fibronectin-positive percentage (fibronectin+, %) was calculated by dividing the fibronectin+ area by the area of the entire field. For each melanoma sample, at least 12 randomly selected fields were analyzed. A minimum of three independent experiments were performed for each quantification of immunostaining.

### Collagen staining

Picrosirius red staining was performed using the Picrosirius Red Stain Kit according to the manufacturer’s instructions (American MasterTech). Briefly, paraffin sections of mouse melanomas were dewaxed, hydrated, and stained with Picrosirius red for 1 hour. After staining, the tissue sections were washed with 1% acetic acid for 1 minute, dehydrated in 100% ethanol, cleared in xylene, and mounted using VectaMount permanent mounting medium for evaluation.

### Quantification of collagen content

Paraffin sections were deparaffinized and rehydrated for collagen content quantification using the Sirius Red/Fast Green Collagen Staining Kit (Chondrex, cat. #9046) following the manufacturer’s instructions. Briefly, after the slide was incubated with the dye solution at room temperature for 30 minutes, the dye solution was removed, and the slide was rinsed with distilled water until the water became colorless. One milliliter of dye extraction buffer was added to each slide to elute the dye from the dyed tissues. Optical absorbances at 540 and 605 nm were measured using a microplate reader. The collagen content was calculated using the following formula: collagen (μg/section) = [OD540 − (OD605 × 0.291)]/0.0378. Three tissue sections per group were used for quantification and statistical analyses.

### Generation of transgene-expressing human CAF lines

To immortalize M27 and M50 cells, Lenti-SV40 lentivirus (ABM, cat. #G203) was purchased from Applied Biological Materials. The immortalized cell lines were named iM27 and iM50. All BRAF and CRAF constructs, including Flag-tagged BRAF (BRAF_OHu28570C_pGenlenti_N-FLAG), Myc-tagged BRAF (HygR_BRAF_OHu28570C_pGenlenti_N-MYC), BRAF-T529N (BRAF-T529N_OHu28570C_pGenlenti_N-FLAG), Flag-tagged CRAF (HygR_RAF1_OHu28584C_pGenlenti_N-Flag), Myc-tagged CRAF (RAF1_OHu28584C_pGenlenti_N-MYC), and CRAF-T421N (RAF1-T421N_OHu28584C_pGenlenti_N-MYC), were designed and cloned by GenScript. All constructs were generated by inserting the respective transgene fragments into the pGenlenti backbone. The respective lentiviruses were produced at the Cincinnati Children’s Hospital Viral Vector Core using the BRAF and CRAF constructs described above.

For lentiviral transduction, 5 × 10^4^ immortalized CAFs were seeded in one well of a six-well plate. When the cells reached 50% confluence, 50 μL of virus was mixed with 450 μL of basal DMEM supplemented with 10 μg/mL polybrene (Santa Cruz, sc-134220). After 16 hours of incubation, the virus medium was replaced with 2 mL of fresh DMEM supplemented with 10% FBS for cell culture. Depending on the antibiotic resistance gene cloned in each lentiviral construct, transduced cells were selected using 10 μg/mL puromycin (Thermo Fisher Scientific, A11138-03), 50 μg/mL hygromycin B (Thermo Fisher Scientific, B-10687010), or 10 μg/mL blasticidin (Santa Cruz, sc-495389). Cells were incubated with antibiotics for 7 days and then transferred to normal DMEM.

### siRNA transfection

Silencer siRNAs targeting ARAF (ID: 153), BRAF (ID: 507), CRAF (ID: 1548), NRAS (ID: 120250), KRAS (ID: 120703), HRAS (ID: 120898), and scramble Silencer siRNA control (cat. #4390843) were purchased from Thermo Fisher Scientific. CAFs expressing BRAF-WT or BRAF (T529N) were transfected with FlexiTube siRNAs targeting BRAF (ID: 673) to silence endogenous BRAF expression. CAFs expressing CRAF-WT or CRAF (T421N) were transfected with FlexiTube siRNAs targeting CRAF (ID: 5894) to silence endogenous CRAF expression. FlexiTube siRNAs targeting the 3′ untranslated region of BRAF and CRAF were obtained from Qiagen.

Briefly, 1 × 10^5^ CAFs were seeded in one well of a six-well tissue culture plate and allowed to grow to approximately 80% confluence. Prior to transfection, culture medium was replaced with fresh DMEM. The cells were then transfected with the indicated siRNAs using Lipofectamine RNAiMAX (Thermo Fisher Scientific, cat. #13778100). For each transfection, a siRNA-Lipofectamine mixture was prepared by incubating 9 μL of Lipofectamine RNAiMAX and 30 pmol of the indicated siRNA in 300 μL of DMEM at room temperature for 5 minutes. The cells in each well were then incubated with siRNA–Lipofectamine mixture for 48 hours at 37°C in a humidified incubator with 5% CO_2_. Afterward, the transfection mixture was replaced with DMEM. To silence NRAS, KRAS, and HRAS expression in CAFs, three siRNAs targeting NRAS, KRAS, and HRAS (30 pmol each as described above) were mixed and incubated with 9 μL of Lipofectamine RNAiMAX in 300 μL DMEM for transfection.

### Pharmacologic treatment of CAFs

To treat CAFs with small-molecule compounds, 5 × 10^3^ cells were seeded in each well of an eight-well Nunc Lab-Tek II chamber slide (Thermo Fisher Scientific, cat. #154534) and cultured for 3 days. The cells were treated as indicated: 2 μmol/L PLX4032 (vemurafenib; Selleck Chemicals, cat. #S1267) for 72 hours; 0.5 μmol/L GSK2118436 (dabrafenib; Selleck Chemicals, cat. #S5069) for 72 hours; 50 nmol/L jasplakinolide (Bio-Techne, cat. #2792) for 2 hours; 500 nmol/L cytochalasin D (CytoD; Bio-Techne, cat. #1233) for 24 hours; 10 μmol/L Y-27632 (Selleck Chemicals, cat. #6390) for 24 hours; 10 μmol/L HA-1077 (Selleck Chemicals, cat. #S1573) for 24 hours; 5 μmol/L GDC0973 (Selleck Chemicals, cat. #S8041) for 24 hours; and 2 μmol/L SCH772984 (Selleck Chemicals, cat. #S7101) for 24 hours. Following treatment, the cells were subjected to IF staining directly on slides.

### Chamber slide IF staining

After treatment, the cells in each chamber were fixed in 4% paraformaldehyde for 10 minutes at room temperature and permeabilized with 0.05% Triton X-100 for 10 minutes on ice for IF staining. After permeabilization, the cells were washed three times with PBS and blocked with 10% BSA for 1 hour at room temperature. Primary antibodies for SUN2 (Millipore, HPA001209, RRID: AB_1080465, 1:200), nesprin-2 (Abcam, ab314872, 1:200), lamin A/C (Proteintech, cat. #10298-1-AP, RRID: AB_2296961, 1:200), lamin A/C (Santa Cruz Biotechnology, cat. #sc-7292, RRID: AB_627875, 1:200), cGAS (Cell signaling, cat. #79978, RRID: AB_2905508, 1:200), acti-stain 555 phalloidin (Cytoskeleton, cat. #PHDH1, 1:200), β-catenin (Thermo Fisher Scientific, cat. #MA1-301, RRID: AB_1070649, 1:500), paxillin (BD Biosciences, cat. #610051, RRID: AB_397463, 1:200), and α-SMA (Thermo Fisher Scientific, cat. #14-9760-82, RRID: AB_2572996, 1:200) were then added to each chamber and incubated overnight at 4°C. The next day, after washing with PBS three times, Alexa Fluor 488- or 594-conjugated secondary antibodies (Thermo Fisher Scientific, cat. #A-11034, RRID: AB_2576217; cat. #A-11032, RRID: AB_2534091, 1:200) were added to the corresponding well for a 1-hour incubation at room temperature. Sections were counterstained with Hoechst 33342 (Cell Signaling Technology, 4082S) at a concentration of 10 μg/mL for 10 minutes at room temperature, mounted with VECTASHIELD Antifade Mounting Medium, and coverslipped. Images were acquired using a Nikon Eclipse 80i fluorescence microscope or Zeiss LSM 710 confocal microscope. Fluorescence intensity was quantified using ImageJ, with identical acquisition settings applied across all experimental conditions.

Nuclear β-catenin intensity was quantified by first defining nuclear regions of interest (ROI) based on Hoechst 33342 fluorescence. The β-catenin signal within each nuclear ROI was measured using ImageJ, and the mean nuclear fluorescence intensity was calculated for each cell. Aberrant SUN2 staining was defined as irregular or mislocalized distribution at the nuclear envelope (NE) or within the nucleus, whereas lamin A/C abnormalities were defined as discontinuous or fragmented staining along the nuclear rim. The percentages of cells with abnormal SUN2 and lamin A/C distribution were quantified by calculating the percentage of nuclei exhibiting aberrant staining relative to the total number of nuclei analyzed. Nesprin-2 cytoplasmic localization was defined as fluorescence signal extending beyond the NE into the cytosol. Disturbed nesprin-2 expression was quantified by measuring and comparing cytosolic nesprin-2 fluorescence intensity across different treatment groups using ImageJ.

### F-actin IF and quantification

CAFs (5 × 10^3^) as indicated were seeded per well in eight-well chamber slides, allowed to adhere overnight, fixed, and stained with Phalloidin Labeling Probes (Cytoskeleton, cat. #PHDH1; 1:200) to visualize F-actin. Nuclei were counterstained with Hoechst 33342. For total F-actin quantification, randomly selected fields were imaged at 20× magnification using a Nikon Eclipse 80i fluorescence microscope with identical acquisition settings across conditions. F-actin fluorescence intensity was measured per cell using ImageJ and normalized to cell area. For actin cap analysis, confocal images were acquired at 40× magnification using a Zeiss LSM 710 confocal microscope. Actin caps were defined as dense F-actin fibers spanning the apical surface of the nucleus. F-actin intensity within actin caps was quantified in ImageJ. To assess 3D organization, Z-stack images were collected at 1-μm intervals through the full nuclear height, as defined by Hoechst staining.

### Quantification of nuclear morphology

First, 0.5 × 10^4^ CAFs as indicated were seeded in one well of an eight-well chamber slide and treated as indicated in each figure. Following treatment, nuclei were stained with Hoechst, and confocal images of randomly selected fields were captured at 40× magnification using a Zeiss LSM 710 confocal microscope. Hoechst-stained images were analyzed using ImageJ software, and the aspect ratio and circularity of individual nuclei were quantified using the particle analysis function in ImageJ.

### Cell stiffness experiments

To evaluate how CAFs respond to stiff matrix environments, CAFs were cultured for 24 hours on soft slides with stiffness values of 0.2 or 50 kPa (Matrigen, SSL8-COL-0.2 and SSL8-COL-50). Cell culture, treatment, and IF staining were performed using the same protocols described above.

### Proximity ligation assay

Proximity ligation assay (PLA) was performed using the Duolink *In Situ* Red Starter Kit (DUO92101, Millipore) according to the manufacturer’s instructions. Briefly, 0.5 × 10^4^ cells were cultured in each well of an eight-well Nunc Lab-Tek II chamber slide. After culturing for 3 days under the indicated conditions, the cells were fixed in 4% paraformaldehyde for 10 minutes at room temperature and permeabilized with 0.1% Triton X-100 in PBS for 10 minutes on ice. Afterward, the cells were blocked with Duolink blocking solution for 1 hour at 37°C and incubated overnight at 4°C with the following primary antibodies: BRAF (Proteintech, cat. #20899-1-AP, RRID: AB_2878760), CRAF (Proteintech, cat. #66592-1-Ig, RRID: AB_2881952), FLAG (Proteintech, cat. #20543-1-AP, RRID: AB_11232216), or MYC (Proteintech, cat. #60003-2-Ig, RRID: AB_2734122). The next day, the slides were washed with wash buffer A and incubated with species-specific PLA probes (PLUS and MINUS) for 1 hour at 37°C. After probe incubation, the cells were washed twice with wash buffer A for 5 minutes each. Ligation-ligase solutions were then added to each well and incubated at 37°C for 30 minutes to ligate the two probes into a circular DNA template. The cells were subsequently washed twice with wash buffer A for 2 minutes each. The circular templates were amplified by adding polymerase in amplification buffer containing nucleotides and fluorescently labeled oligonucleotides, resulting in distinct fluorescent spots. The slides were then mounted with Duolink *in situ* mounting medium containing DAPI for 30 minutes and imaged using a Nikon Eclipse 80i fluorescence microscope. PLA signals were quantified using ImageJ as either the average dot intensity per 40× field or number of dots per cell.

### Co-immunoprecipitation

Co-immunoprecipitation (co-IP) was performed using a Pierce Crosslink Immunoprecipitation Kit according to the manufacturer’s instructions (Thermo Fisher Scientific). Briefly, 10 μg of Myc-tag antibody (Proteintech, cat. #60003-2-Ig, RRID: AB_2734122) or mouse IgG (Thermo Fisher Scientific, cat. #31903) was added to a spin column containing 20 μL of resin slurry in a 1.5-mL collection tube. The antibodies and beads were incubated at room temperature with gentle rotation for 1 hour. After incubation, the spin columns were centrifuged, and the antibody-bound resin was subsequently rinsed with 1× coupling buffer three times to remove unbound antibodies. A 2.5 mmol/L disuccinimidyl suberate crosslinker solution was added to crosslink the antibody to the resin. Subsequently, 1 mg of protein extracted from the cells coexpressing Flag-tagged CRAF and Myc-tagged CRAF with or without PLX4032 treatment was added to a spin column and incubated with rotation at 4°C overnight. After incubation, the column was washed three times with 1× TBS and one time with 1× conditioning buffer. The samples were eluted in 50 μL of elution buffer. The eluate was boiled with sample buffer at 95°C for 5 minutes for Western blotting.

### Quantitative real-time PCR assay

As indicated, 2 × 10^5^ cells were seeded in one 6-cm culture dish and treated with either DMSO or BRAFi for 3 days. Total RNA was isolated from cell pellets using the PureLink RNA Mini Kit (Thermo Fisher Scientific, cat. #12183020) according to the manufacturer’s instructions. RNA concentration was measured using a NanoDrop one spectrophotometer (Thermo Fisher Scientific). cDNA was synthesized by reverse transcription using the RevertAid First Strand cDNA Synthesis Kit (Thermo Fisher Scientific, cat. #K1622). Quantitative real-time PCR was performed using SYBR Green Master Mix (Thermo Fisher Scientific, cat. #A25742) on a StepOnePlus Real-Time PCR System (Applied Biosystems). Primers for CXCL1, CXCL2, CXCL12, IL1A, IL1B, IL6, IL8, IL33, COL1A1, COL1A2, VEGFA, TAGLN, ACTN1, FN1, and CYP1B1 were purchased from RealTimePrimers.com. Relative gene expression levels were normalized to ACTB. Data are representative of three independent experiments.

### Nuclear–cytoplasmic fractionation

Nuclear–cytoplasmic fractionation was performed using the Nuclear and Cytoplasmic Extraction Reagents (Thermo Fisher Scientific, cat. #78833) according to the manufacturer’s instructions. Briefly, 2 × 10^5^ cells were seeded in 10-cm culture dishes and treated with either DMSO or 2 μmol/L PLX4032 for 3 days. Cells were then harvested, and the resulting pellets were subjected to fractionation for Western blotting analyses.

### Western blotting

For Western blotting, 2 × 10^5^ cells as indicated were seeded in one 10-cm culture dish and treated as described above for 3 days. After treatment, proteins were extracted using RIPA buffer supplemented with ethylenediaminetetraacetic acid (EDTA)-free Halt Protease and Phosphatase Inhibitor Cocktail (Thermo Fisher Scientific, cat. #78443). Briefly, cells were lysed in RIPA buffer on ice for 30 minutes with brief vortexing every 10 minutes. The lysates were then centrifuged at 15,000 × *g* for 15 minutes at 4°C, and the supernatants were collected and transferred to fresh tubes. Protein was quantified using the Bicinchoninic Acid protein assay (Thermo Fisher Scientific, cat. #A55860).

Twenty μg of protein was loaded per lane and run on a 10% to 12% SDS-PAGE gel and subsequently transferred to a nitrocellulose membrane. The membranes were blocked with 5% fat-free milk or 5% BSA in TBST for 1 hour at room temperature and then incubated with the following primary antibodies: ARAF (Cell Signaling Technology, cat. #4432, RRID: AB_330813, 1:1,000), BRAF (Proteintech, cat. #20899-1-AP, RRID: AB_2878760, 1:1,000), CRAF (Proteintech, cat. #66592-1-Ig, RRID: AB_2881952, 1:1,000), CRAF (Proteintech, cat. #26863-1-AP, RRID: AB_2880660, 1:1,000), phospho-BRAF (Cell Signaling Technology, cat. #2696, RRID: AB_390721, 1:1,000), phospho-CRAF (Cell Signaling Technology, cat. #9427, RRID: AB_2067317, 1:1,000), Flag (Proteintech, cat. #20543-1-AP, RRID: AB_11232216, 1:1,000), MYC (Proteintech, cat. #60003-2-Ig, RRID: AB_2734122, 1:1,000), MYC (Cell Signaling Technology, cat. #2278, RRID: AB_490778, 1:1,000), KRAS (Proteintech, cat. #12063-1-AP, RRID: AB_878040, 1:1,000), HRAS (Proteintech, cat. #18295-1-AP, RRID: AB_2121046, 1:1,000), NRAS (Proteintech, cat. #10724-1-AP, RRID: AB_2154209, 1:1,000), ERK1/2 (Cell Signaling Technology, cat. #9107, RRID: AB_10695739, 1:1,000), phospho-ERK (Cell Signaling Technology, cat. #4370, RRID: AB_2315112, 1:1,000), MYPT1 (Proteintech, cat. #22117-1-AP, RRID: AB_11183753, 1:1,000), phospho-MYPT1 (Millipore, cat. #ABS45, RRID: AB_11212365), SUN2 (Millipore, HPA001209, RRID: AB_1080465, 1:1,000), nesprin-2 (Abcam, ab314872, 1:1,000), lamin A/C (Proteintech, cat. #10298-1-AP, RRID: AB_2296961, 1:1,000), lamin B1 (Proteintech, cat. #12987-1-AP, RRID: AB_2136290, 1:1,000), and Histone H3 (Proteintech, cat. #17168-1-AP, RRID: AB_2716755, 1:1,000) in blocking buffer. After washing three times with TBST, the membranes were then incubated with IRDye 800CW donkey anti-rabbit IgG secondary antibody (LI-COR, 926–32213, 1:2,000) or IRDye 680RD donkey anti-mouse IgG secondary antibody (LI-COR, 926–68072, 1:2,000) at a 1:2,000 dilution in blocking buffer and washed three times with TBST. The membranes were imaged using an Odyssey CLx imaging system (LI-COR, model #9140).

### Quantification and statistical analysis

Data were analyzed using the GraphPad Prism 10 software package (GraphPad Software) and expressed as the mean ± SD. Comparison between two independent groups was performed using unpaired, two-tailed Student *t* tests. Comparisons among more than two groups were conducted using one-way analysis of variance, followed by Tukey *post hoc* multiple comparison test. A *P* value < 0.05 was considered statistically significant.

### Ethical approval

The Institutional Animal Care and Use Committee of the University of Cincinnati approved all experimental procedures involving mice under protocol 22-08-19-01.

## Results

### BRAFi induce nuclear deformation in CAFs

**Figure 1.**
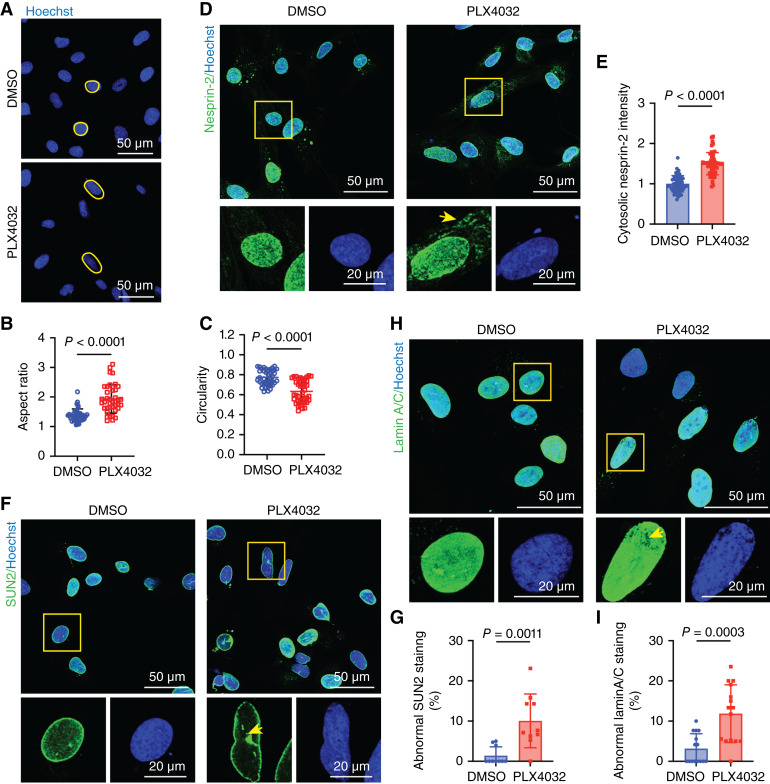
BRAFi induce nuclear deformation in CAFs. **A,** Representative confocal images showing nuclei stained with Hoechst in iM27 cells with or without PLX4032 treatment. Yellow circles indicate the approximate nuclear boundaries of representative cells under the different conditions. Scale bars, 50 μm. **B** and **C,** Scatter dot plots showing the nuclear morphologic parameters in iM27 cells with or without PLX4032 treatment. Nuclear aspect ratio (**B**) and circularity (**C**) were measured from confocal images and quantified using ImageJ. Data are presented as the mean ± SD (*n* = 40–46 nuclei per group). **D,** Confocal images showing immunostaining of nesprin-2 in iM27 cells with or without PLX4032 treatment. Small panels show single-channel fluorescence images of representative cells highlighted by yellow boxes in the larger images above. Abnormal nesprin-2 distribution in PLX4032-treated iM27 cells is indicated by a yellow arrow. Scale bar as indicated. **E,** Quantification of cytosolic nesprin-2 intensity in iM27 cells under indicated conditions. Data are presented as the mean ± SD (*n* = 49–57 cells). **F,** Confocal images showing immunostaining of SUN2 in iM27 cells with or without PLX4032 treatment. Small panels show single-channel fluorescence images of representative cells highlighted by yellow boxes in the larger images above. Disorganized SUN2 distribution pattern in PLX4032-treated iM27 cells is indicated by a yellow arrow. Scale bar as indicated. **G,** Quantification of the percentage of iM27 cells showing abnormal staining of SUN2 under indicated conditions. Data are presented as the mean ± SD (*n* = 10–15 random 40× fields). **H,** Confocal images showing immunostaining of nuclear lamin A/C in iM27 cells with or without PLX4032 treatment. Small panels show single-channel fluorescence images of representative cells highlighted by yellow boxes in the larger images above. Disorganized lamin A/C distribution in PLX4032-treated iM27 cells is indicated by a yellow arrow. Scale bar as indicated. **I,** Quantification of the percentage of iM27 cells showing disturbed lamin A/C nuclear organization under indicated conditions. Data are presented as the mean ± SD (*n* = 10–15 random 40× fields).

CAFs can translate BRAFi treatment and mechanical signals into adaptive responses that promote CAF activation and tumor progression (Supplementary Fig. S1; ref. 7). However, the precise mechanisms by which BRAFi drive CAF activation and cross-talk with mechanical signaling pathways remain to be fully elucidated. Nuclear morphologic changes are a known mechanism by which cells regulate gene expression (30). To understand how CAFs adapt to BRAFi, we first examined nuclear morphology in CAFs after treatment with PLX4032 (Fig. 1; Supplementary Fig. S2) and GSK2118436 (Supplementary Fig. S3). As shown in Fig. 1A, the nuclei in many BRAFi-treated CAFs became elongated. The average nuclear aspect ratio in BRAFi-treated CAFs, which was calculated by dividing the length of the longest axis of the nucleus by the length of its shortest axis, was ∼1.94, compared with ∼1.39 in DMSO-treated CAFs (Fig. 1B), suggesting that BRAFi induced nuclear elongation in CAFs. Furthermore, nuclear circularity, which represents the roundness of a nucleus, was reduced in BRAFi-treated CAFs to ∼0.63 from ∼0.77 in DMSO-treated CAFs (Fig. 1C). These observations indicate that BRAFi caused the nuclei in CAFs to undergo morphologic deformation.

Nuclear shape and positioning are regulated by the linker of the nucleoskeleton and cytoskeleton (LINC) complex, which connects the nucleus (nucleoskeleton) to the cytoskeleton (31). Alterations in the LINC complex in BRAFi-treated CAFs were examined by IF staining of two core LINC components, nesprin-2 and SUN2. Nesprin-2 is anchored in the outer nuclear membrane and interacts with actin filaments, thereby joining the cytoskeleton to the nucleoskeleton (32). As shown in Fig. 1D, nesprin-2 in CAFs extended into the cytoplasm. This structural arrangement enables the LINC complex to sense and transmit mechanical and associated signaling cues from the cytoplasm to the nucleus. Interestingly, BRAFi treatment increased cytoplasmic localization of nesprin-2 surrounding the nucleus (Fig. 1E). SUN2 is a membrane-embedded protein located in the inner nuclear membrane, where it interacts with nuclear lamins such as lamin A/C (33). BRAFi-treated CAFs presented aberrant nuclear distribution of SUN2, suggesting disruption of the LINC complex and possible compromise of NE integrity (Fig. 1F and G). As the organization of nesprin-2 and SUN2 was disrupted, we next investigated whether there were changes in nuclear lamins, as they are directly connected to the inner nuclear membrane and are essential structural proteins (34). Discontinuous staining of lamin A/C in BRAFi-treated CAFs suggested the disruption of the nuclear lamin structure (Fig. 1H and I). The findings imply that changes in the LINC complex may contribute to the nuclear deformation in BRAFi-treated CAFs.

### Actin-mediated cytoskeletal remodeling drives BRAFi-induced nuclear deformation in CAFs

Actin polymerization, particularly in the perinuclear region, plays an important role in shaping the nucleus by transmitting mechanical forces through the LINC complex (35). Increased actin polymerization may therefore exert mechanical stress on the nucleus and potentially induce nuclear rupture. To assess NE rupture, we examined the cytosolic DNA sensor cGAS, which can rapidly accumulate at sites of NE rupture and bind exposed chromatin (36). Quantification showed that PLX4032 treatment did not increase cGAS accumulation at the NE in CAFs (Supplementary Fig. S4A–S4D). Consistently, cytoplasmic and nuclear fractionation analyses revealed no detectable leakage of nuclear proteins, including lamin A/C, lamin B, and the chromatin marker Histone H3, into the cytosol following PLX4032 treatment in iM27 and iM50 cells (Supplementary Fig. S4E and S4F). These results suggest that nuclear deformation induced by BRAFi in CAFs does not lead to significant NE rupture.

To understand whether the BRAFi-induced changes in the LINC complex and nuclear elongation observed in CAFs were caused by actin-mediated nuclear force, we investigated actin filament organization in PLX4032-treated CAFs. As shown in Supplementary Fig. S5A and S5B, the abundance of polymerized actin filaments was greater in CAFs than in normal dermal fibroblasts. However, the actin structures were much more prominent in CAFs treated with PLX4032 (Supplementary Fig. S5C and S5D), suggesting that BRAFi induced cytoskeletal remodeling in CAFs. We focused on the perinuclear actin cap, a specialized subset of actin filaments that span the nucleus (37), because it is directly anchored to LINC complexes on the NE. Z-stack confocal imaging revealed prominent assembly of actin cap structures in PLX4032-treated CAFs, in which F-actin aligned above the nucleus in a dome-like structure and colocalized with nuclear staining, as indicated by the yellow arrow (Supplementary Fig. S5E).

These observations suggest that enhanced actin cytoskeletal remodeling induced by BRAFi generates mechanical forces that contribute to nuclear deformation in CAFs. To test this hypothesis, we treated CAFs with jasplakinolide (jaspla), an actin polymerization inducer known to promote cytoskeletal remodeling (38). As shown in Supplementary Fig. S6A and S6B, actin polymerization and the formation of perinuclear actin caps were significantly increased in jaspla-treated CAFs. Furthermore, jaspla treatment also induced nuclear deformation and disorganized SUN2 distribution (Supplementary Fig. S6C–S6F). These data support the idea that increased cytoskeletal remodeling contributes directly to nuclear deformation in CAFs.

**Figure 2.**
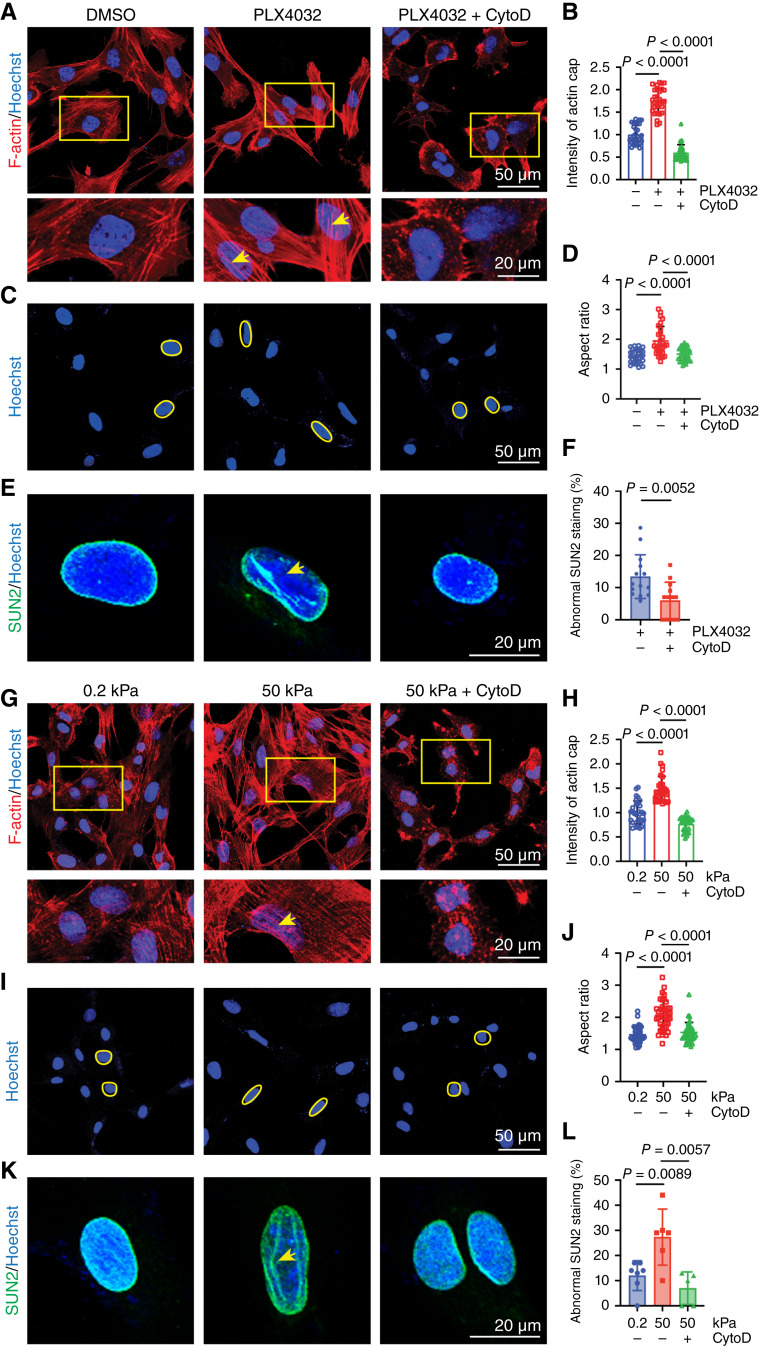
BRAFi and ECM stiffness converge on actin-mediated cytoskeletal remodeling to drive nuclear deformation in CAFs. **A,** Confocal images showing F-actin in iM27 cells treated with DMSO, PLX4032, or the combination of PLX4032 and CytoD. Representative individual cells shown in the insets correspond to the cells highlighted by yellow boxes in the larger images above. Actin caps are indicated by yellow arrows. Scale bar as indicated. **B,** Quantification of actin cap intensity from (**A**) using ImageJ. Data are presented as the mean ± SD (*n* = 30 nuclei per group). **C,** Representative confocal images of Hoechst-stained nuclei in iM27 cells treated with DMSO, PLX4032, or the combination of PLX4032 and CytoD. Yellow circles indicate the approximate nuclear boundaries. Scale bars, 50 μm. **D,** Scatter dot plots showing nuclear morphological changes in iM27 cells under three different conditions. Nuclear aspect ratio was analyzed and quantified from confocal images using ImageJ. Data are presented as the mean ± SD (*n* = 26–39 nuclei per group). **E,** Confocal images showing SUN2 staining in iM27 cells treated with DMSO, PLX4032, or the combination of PLX4032 and CytoD. Yellow arrows indicate the disorganized SUN2 distribution in iM27 cells treated with PLX4032 alone. Scale bars, 20 μm. **F,** Quantification of the percentage of iM27 cells exhibiting abnormal SUN2 staining. Data are presented as the mean ± SD (*n* = 13–14 random 40× fields). **G,** Confocal images showing F-actin in iM27 cells cultured on soft slides with a stiffness of 0.2 kPa (left), stiff slides with a stiffness of 50 kPa (middle), and stiff slides with a stiffness of 50 kPa and CytoD treatment (right). Representative individual cells shown in the insets correspond to the cells highlighted by yellow boxes in the larger images above. Actin caps are indicated by yellow arrows. Scale bar as indicated. **H,** Quantification of actin cap intensity using ImageJ. Data are presented as the mean ± SD (*n* = 25–30 nuclei per group). **I,** Representative confocal images of Hoechst-stained nuclei in iM27 cells cultured on soft slides with a stiffness of 0.2 kPa (left), stiff slides with a stiffness of 50 kPa (middle), and stiff slides with a stiffness of 50 kPa and CytoD treatment (right). Yellow circles indicate the approximate nuclear boundaries. Scale bars, 50 μm. **J,** Scatter dot plots showing nuclear morphological changes in iM27 cells under three different conditions. Nuclear aspect ratio was analyzed and quantified from confocal images using ImageJ. Data are presented as the mean ± SD (*n* = 40 nuclei per group). **K,** Confocal images showing SUN2 staining in iM27 cells cultured on soft slides with a stiffness of 0.2 kPa (left), stiff slides with a stiffness of 50 kPa (middle), and stiff slides with a stiffness of 50 kPa and CytoD treatment (right). Disorganized SUN2 distribution in iM27 cells cultured on 50 kPa slides is indicated by yellow arrows. Scale bars, 20 μm. **L,** Quantification of the percentage of iM27 cells exhibiting abnormal SUN2 staining. Data are presented as the mean ± SD (*n* = 5–7 random 40× fields).

We then investigated whether inhibiting actin polymerization could reverse the changes in nuclear shape induced by PLX4032. To this end, CAFs were treated with PLX4032 alone or in combination with CytoD, an actin polymerization inhibitor (39). F-actin staining confirmed a reduction in actin fiber polymerization and actin cap structure formation in CAFs treated with PLX4032 + CytoD compared with those treated with PLX4032 (Fig. 2A and B), suggesting that CytoD effectively disrupted actin filament assembly driven by PLX4032. Consistent with this, PLX4032-induced nuclear deformation and aberrant SUN2 distribution were significantly reversed by CytoD (Fig. 2C–F).

### Matrix stiffness recapitulates BRAFi-induced actin remodeling and nuclear deformation in CAFs

Given that increased tissue stiffness is a classic characteristic of solid tumors, we tested whether elevated matrix stiffness promotes actin polymerization and nuclear deformation in CAFs. CAFs were cultured on soft (0.2 kPa) or stiff (50 kPa) substrates representing the physiologic extremes of the skin mechanical microenvironment. The 0.2 kPa matrix approximates homeostatic dermal ECM stiffness (∼0.1 to 1 kPa), whereas 50 kPa reflects the pathologically stiffened matrix observed in fibrosis, chronic inflammation, or tumor-associated desmoplasia (40–42). In line with the effects of PLX4032 stimulation, increased ECM stiffness induced marked increases in actin polymerization and actin cap formation in CAFs cultured on stiff substrates compared with soft substrates. This stiffness-induced cytoskeletal remodeling was abrogated by CytoD, mirroring its effect upon PLX4032 stimulation (Fig. 2G and H). Increased ECM stiffness also elevated the nuclear aspect ratio and disrupted SUN2 organization, suggesting that the nuclei were deformed. CytoD reversed these stiffness-induced nuclear changes, reducing the aspect ratio to ∼1.53 (Fig. 2I–L). These findings suggest that ECM stiffness elicits nuclear deformation by driving cytoskeletal remodeling, indicating a common force transmission mechanism in CAFs that is shared with BRAF inhibition.

### Actin-mediated nuclear deformation is associated with β-catenin nuclear accumulation in CAFs

Next, we aimed to understand how nuclear dynamics translates into molecular signals in CAFs. Previously, we reported that BRAFi induce Wnt-independent activation of nuclear β-catenin signaling in CAFs (7). β-catenin is a dual-function protein that participates in cell adhesion at the plasma membrane and mediates signaling by shuttling between the cytoplasm and nucleus. Confocal imaging revealed increased nuclear β-catenin accumulation in jaspla-treated CAFs, similar to that observed in PLX4032-treated CAFs (Supplementary Fig. S7A–S7D), indicating an association between nuclear deformation and β-catenin nuclear accumulation. In contrast, CytoD treatment suppressed nuclear β-catenin accumulation in PLX4032-treated CAFs. Similarly, the increased nuclear accumulation of β-catenin in CAFs cultured on stiff substrates compared with soft substrates was markedly reduced upon CytoD treatment (Supplementary Fig. S7E and S7F). Collectively, these data suggest that actin-mediated nuclear remodeling is associated with β-catenin nuclear accumulation driven by both increased matrix stiffness and BRAFi.

### Constitutive β-catenin activation in fibroblasts enhances CAF-like features and melanoma growth

Nuclear β-catenin is crucial for regulating the expression of genes that are essential for cell proliferation, differentiation, and survival (43). As β-catenin accumulates in the nucleus in response to various stimuli, including BRAFi and stiff substrates, we wanted to understand the biological outcome of increased nuclear β-catenin in fibroblasts and melanoma development. As shown in Supplementary Fig. S8A and S8B, fibroblasts overexpressing a constitutively active form of β-catenin (*Col1α2-CreER*^*T2*^; *Rosa-rtTA*; *TetO-ΔN-β-catenin*, hereafter ΔN-β-catenin) displayed higher nuclear β-catenin expression than control fibroblasts expressing endogenous β-catenin (*Col1α2-CreER*^*T2*^; *Rosa-rtTA*, hereafter control). These ΔN-β-catenin–overexpressing fibroblasts exhibited increased proliferation (Supplementary Fig. S8C) and elevated expression of α-SMA, which is a well-established CAF marker (Supplementary Fig. S8D and S8E). Furthermore, the expression and distribution of paxillin and F-actin were upregulated in ΔN-β-catenin–expressing fibroblasts (Supplementary Fig. S8F–S8I). As changes in the actin cytoskeleton and scaffolding proteins such as paxillin are often linked to cell contractility, we assessed their contractile capacity using a gel contraction assay and confocal reflection microscopy (Supplementary Fig. S8J–S8N). The results showed that ΔN-β-catenin–expressing CAFs exhibited increased contractile activity. Importantly, as noted above, increased actin polymerization further amplifies nuclear deformation and β-catenin accumulation, supporting a potential reciprocal feed-forward loop that can continuously enhance the biological properties of fibroblasts.

**Figure 3.**
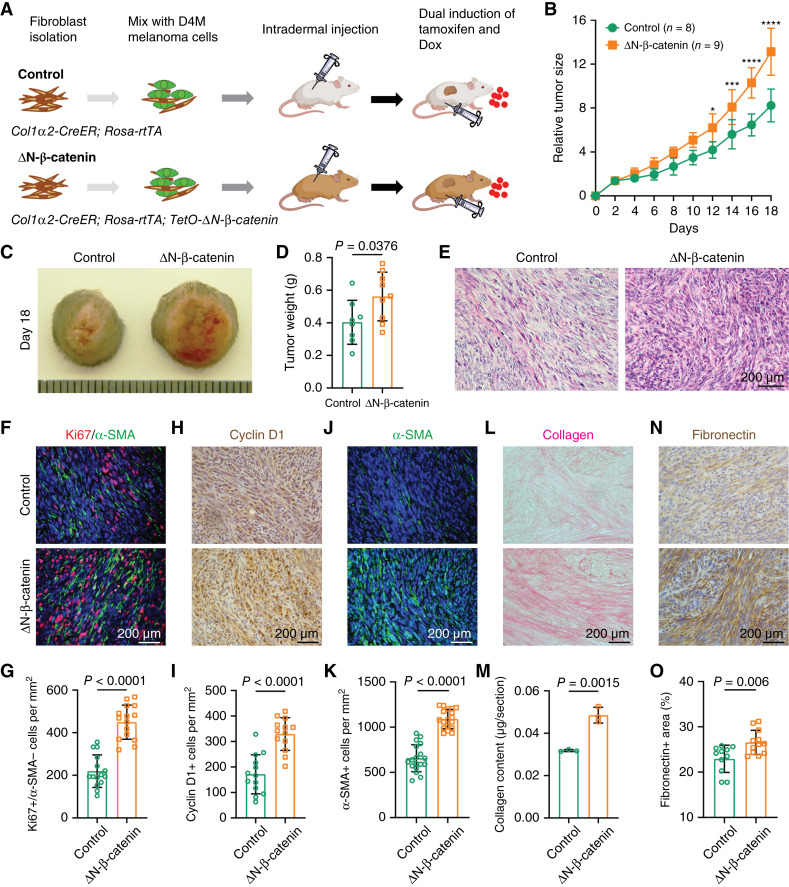
CAF β-catenin activation accelerates melanoma progression *in vivo*. **A,** Schematic illustration of the melanoma mouse model used to study the *in vivo* effects of β-catenin–overexpressing fibroblasts on melanoma progression. D4M melanoma cells (green) were mixed with uninduced control fibroblasts (*Col1α2-CreER*; *Rosa-rtTA*, control melanoma) or mutant fibroblasts (*Col1α2-CreER*; *Rosa-rtTA*; *TetO-ΔN-β-catenin*, ΔN-β-catenin melanoma) and injected intradermally into the flanks of B6 mice. Tumors were allowed to grow until they reached approximately 62.5 mm^3^ in volume. At this point (designated day 0), tamoxifen and Dox were administered to induce β-catenin overexpression in mutant fibroblasts. Tumor growth was subsequently monitored every other day until the end point. **B,** Tumor sizes were measured every other day and compared between control melanomas and ΔN-β-catenin melanomas (*n* = 8–9). **C,** Representative images of control melanomas and ΔN-β-catenin melanomas on day 18. **D,** Comparison of tumor weight between control melanomas and ΔN-β-catenin melanomas on day 18. Each data point represents the weight of an individual tumor. The data are presented as the mean ± SD (*n* = 8–9). **E,** H&E-stained control and ΔN-β-catenin melanoma tissue sections captured using a light microscope. **F,** Representative images showing α-SMA and Ki67 IF staining in control and ΔN-β-catenin melanoma tissue sections as indicated. Nuclei were counterstained with DAPI (blue). **G,** Quantitative comparison of the numbers of α-SMA− cells that were Ki67+ in each group. Each data point represents the number of α-SMA−, Ki67+ cells per mm^2^ counted in each melanoma sample. *n* = 15. **H,** Representative images showing cyclin D1 staining in control and ΔN-β-catenin melanoma tissue sections as indicated by IHC. **I,** Quantitative comparison of the numbers of cyclin D1+ cells in each group. Each data point represents the number of cyclin D1+ cells per mm^2^ counted in each melanoma sample. *n* = 13. **J,** Representative images showing α-SMA staining in control and ΔN-β-catenin melanoma tissue sections as indicated. Nuclei were counterstained with DAPI (blue). **K,** Quantitative comparison of the number of α-SMA+ cells in each group. Each data point represents the number of α-SMA+ cells per mm^2^ counted in each melanoma sample. *n* = 18. **L,** Representative images showing collagen staining in melanoma tissue sections as indicated. **M,** Quantitative comparison of collagen contents in control and ΔN-β-catenin melanoma tissue sections by collagen extraction and colorimetric measurement. *n* = 3. **N,** IHC images showing the expression of the ECM protein fibronectin in control and ΔN-β-catenin melanoma tissue sections as indicated. **O,** Quantitative comparison of the percentages of the surface areas occupied by fibronectin+ fibroblasts measured in control and ΔN-β-catenin melanoma tissue sections. Eleven to twelve random fields from three melanoma pairs were counted. *n* = 11–12. For all the staining images, the scale bar represents 200 μm. In all the statistical graphs, the data are presented as the mean ± SD. *, *P* ≤ 0.05; ***, *P* ≤ 0.001; ****, *P* ≤ 0.0001.

To investigate the importance of ΔN-β-catenin–expressing CAFs in *in vivo* melanoma progression, we coinjected fibroblasts and BRAF-mutant D4M melanoma cells intradermally into B6 mice to induce melanoma formation (Fig. 3A). Melanomas containing ΔN-β-catenin–expressing CAFs (ΔN-β-catenin melanomas) grew faster than those containing WT CAFs at each time point (Fig. 3B). On day 18, when tumors were collected, melanomas containing ΔN-β-catenin–expressing CAFs were larger and had greater tumor weight than control melanomas (Fig. 3C and D), suggesting that upregulated β-catenin activity in CAFs promoted melanoma growth *in vivo*. H&E staining revealed that the structure of the ΔN-β-catenin melanomas was more compact with fewer intercellular spaces than that of the control tumors (Fig. 3E). To confirm this observation, we assessed the expression of two major proliferation markers, Ki67 and cyclin D1. As shown in Fig. 3F and G, the number of Ki67+ melanoma cells (α-SMA−, shown in Supplementary Fig. S9) was notably increased in ΔN-β-catenin melanomas, suggesting that upregulated β-catenin activity in CAFs promoted the proliferative activity of BRAF-mutant melanoma cells. In line with this observation, increased expression of cyclin D1 was detected in ΔN-β-catenin melanomas (Fig. 3H and I).

Increased α-SMA expression was observed in these melanomas, indicating that upregulated β-catenin activity drove CAF activation (Fig. 3J and K), which was consistent with the *in vitro* data showing that β-catenin overexpression promoted fibroblast proliferation (Supplementary Fig. S8C). CAFs are the major producers of ECM components, such as fibronectin and collagen, which play critical roles in remodeling the TME and promoting tumor malignancy. Increased fibronectin and collagen deposition were observed in ΔN-β-catenin melanomas (Fig. 3L–O). These findings suggest that increased nuclear β-catenin, observed in response to BRAFi or stiff stroma, is associated with CAF-like activation and may contribute to matrix remodeling and melanoma progression.

### RAF dimerization and transactivation underlie BRAFi-induced β-catenin nuclear accumulation in CAFs

**Figure 4.**
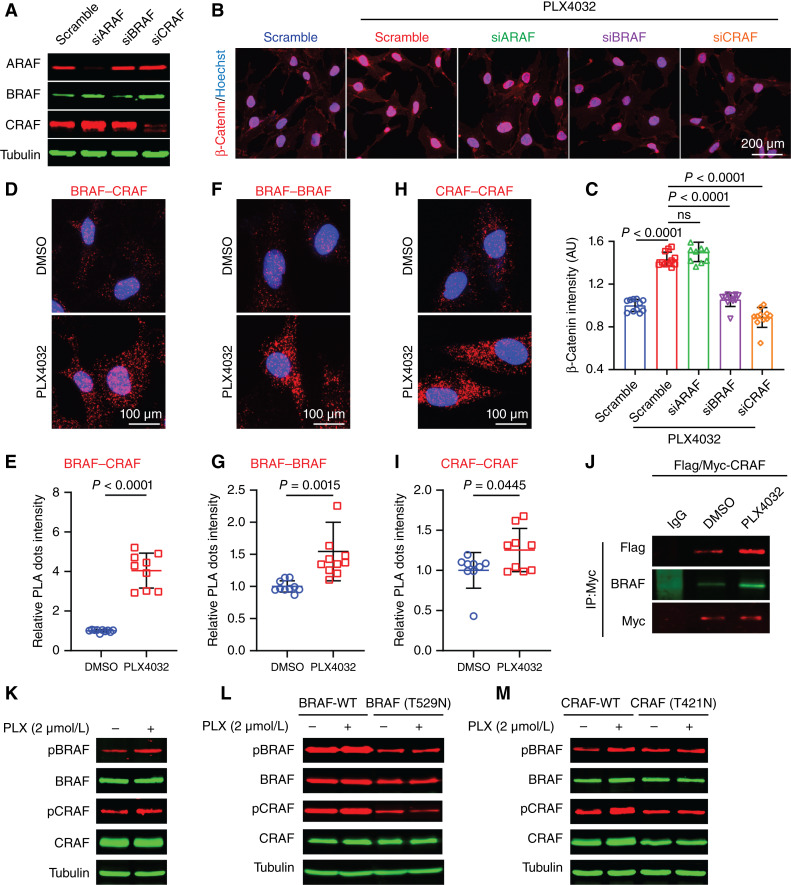
BRAFi promote BRAF/CRAF dimerization and transactivation to drive β-catenin nuclear accumulation in CAFs. **A,** Western blot confirming effective silencing of ARAF, BRAF, or CRAF expression in iM27 cells using corresponding siRNAs. **B,** Representative fluorescence microscopy images showing nuclear β-catenin expression in iM27 cells transfected with scramble siRNA or siRNAs that deplete ARAF, BRAF, or CRAF expression under PLX4032 treatment. iM27 transfected with scramble siRNA without PLX4032 treatment were used as a control. Scale bars, 200 μm. **C,** Quantitative comparison of nuclear β-catenin intensity in iM27 and RAF-depleted iM27 cells as shown in (**B**) with or without PLX4032 treatment using ImageJ. Data are presented as the mean ± SD (*n* = 11 randomly selected 20× fields per group). **D,** Representative PLA images showing BRAF–CRAF heterodimerization in DMSO- and PLX4032-treated iM27 cells. Red dots indicate BRAF–CRAF dimers. Scale bars, 100 μm. **E,** Quantification of BRAF–CRAF PLA signals comparing PLX4032-treated iM27 cells with DMSO-treated iM27 cells. Data are presented as the mean ± SD (*n* = 9–10 randomly selected 40× fields per group). **F,** Representative PLA images showing BRAF–BRAF homodimerization in DMSO- and PLX4032-treated iM27 cells. The red dots indicate BRAF–BRAF dimers. **G,** Quantification of BRAF–BRAF PLA signals comparing PLX4032-treated iM27 cells with DMSO-treated iM27 cells. Data are presented as the mean ± SD (*n* = 9–10 randomly selected 40× fields per group). **H,** Representative PLA images showing CRAF–CRAF homodimerization in DMSO- and PLX4032-treated iM27 cells. The red dots indicate CRAF–CRAF dimers. **I,** Quantification of CRAF–CRAF PLA signals comparing PLX4032-treated iM27 cells with DMSO-treated iM27 cells. Data are presented as the mean ± SD (*n* = 9–10 randomly selected 40× fields per group). **J,** Western blot analysis showing increased CRAFCRAF and CRAF–BRAF interactions in PLX4032-treated iM27 cells compared with those in DMSO-treated iM27 cells. Co-IP was performed using an anti-Myc antibody to pull down interacting proteins in iM27 cells coexpressing Myc-tagged and Flag-tagged CRAF. **K–M,** Western blot analysis of phosphorylated BRAF at Ser445 and phosphorylated CRAF at Ser338 in iM27 cells treated with DMSO or PLX4032. **K,** Endogenous BRAF and CRAF phosphorylation in iM27 cells. **L,** BRAF-deficient iM27 cells following lentiviral reexpression of WT BRAF or kinase domain-dead mutant BRAF (T529N). **M,** CRAF-deficient iM27 cells following lentiviral reexpression of WT CRAF or kinase domain-dead mutant CRAF (T421N).

We then investigated how BRAFi treatment reorganizes the cytoskeleton to promote nuclear deformation and β-catenin nuclear accumulation in CAFs. The RAF kinase family includes three serine/threonine-specific protein kinases, ARAF, BRAF, and CRAF (44). We used siRNAs to selectively silence each RAF kinase to determine which RAF isoforms mediate the response to PLX4032 in CAFs (Fig. 4A). As shown in Fig. 4B and C, ARAF depletion had no significant effect on PLX4032-induced nuclear accumulation of β-catenin compared with that in scramble siRNA-transfected controls. In contrast, loss of either BRAF or CRAF markedly suppressed β-catenin nuclear accumulation in response to PLX4032. These findings were confirmed in the additional CAF lines (Supplementary Fig. S10A–S10C). Together, these observations indicate that BRAF and CRAF, but not ARAF, are involved in mediating the nuclear accumulation of β-catenin driven by BRAFi in CAFs.

The activation of RAF isoforms is intrinsically linked to their ability to form dimers, either homodimers (e.g., BRAF–BRAF or CRAF–CRAF) or heterodimers (e.g., BRAF–CRAF), depending on the cellular context. To understand how BRAFi induce paradoxical RAF activation in CAFs, we first examined RAF dimerization using PLA. PLA revealed greater BRAF–CRAF heterodimer formation in PLX4032-treated CAFs than in control cells (Fig. 4D and E; Supplementary Fig. S10D and S10E). To investigate RAF homodimer formation in CAFs, we coexpressed Flag-tagged BRAF and Myc-tagged BRAF to identify BRAF–BRAF homodimerization, and in a separate set of cells, coexpressed Flag-tagged CRAF and Myc-tagged CRAF to assess CRAF–CRAF homodimerization. PLX4032 treatment notably enhanced the formation of both BRAF–BRAF and CRAF–CRAF homodimers (Fig. 4F–I). Additionally, the formation of BRAF and CRAF dimers was confirmed using another BRAFi, GSK2118436 (Supplementary Fig. S10F–S10I). Co-IP using an anti-Myc antibody further confirmed increased CRAF homodimerization and heterodimerization in PLX4032-treated CAFs coexpressing Flag- and Myc-tagged CRAF (Fig. 4J). Furthermore, phosphorylation of BRAF at Ser445 and CRAF at Ser338, established markers of RAF activation, was elevated in CAFs following PLX4032 treatment (Fig. 4K). Together, these findings suggest that BRAFi promote RAF activation in CAFs by increasing RAF dimerization and phosphorylation.

In melanoma cells harboring the BRAF V600E mutation, PLX4032 binds to the kinase domain of the mutant BRAF to block its enzymatic activity. However, whether PLX4032 binding to the kinase domains of WT BRAF and CRAF is required for increased RAF dimerization remains unclear. To address this, we generated two CAF lines expressing mutant BRAF and CRAF, respectively, carrying gatekeeper mutations in their kinase domains that prevent BRAFi from accessing the corresponding ATP-binding pockets: one BRAF-deficient line expressing BRAF (T529N) and another CRAF-deficient line expressing CRAF (T421N; Supplementary Fig. S11A and S11D; ref. 45). The depletion of endogenous BRAF or CRAF significantly reduced PLX4032-induced nuclear β-catenin accumulation in control CAFs (siBRAF and siCRAF). A similar nuclear β-catenin reduction was observed in CAFs expressing the gatekeeper mutants BRAF (siBRAF + T529N) and CRAF (siCRAF + T421N) but not in CAFs expressing WT BRAF (siBRAF + BRAF-WT) or CRAF (siCRAF + CRAF-WT; Supplementary Fig. S11B, S11C, S11E, and S11F). Notably, PLX4032 and GSK2118436 failed to induce BRAF–CRAF heterodimer formation in CAFs expressing either BRAF (T529N) or CRAF (T421N; Supplementary Fig. S12). As expected, in BRAF- or CRAF-depleted CAFs reexpressing the corresponding gatekeeper mutant, PLX4032 failed to increase BRAF Ser445 or CRAF Ser338 phosphorylation, indicating that BRAFi access to the BRAF or CRAF kinase domain is required for RAF dimerization and activation (Fig. 4L and M).

### RAS is required for BRAFi-induced RAF dimerization and phosphorylation

**Figure 5.**
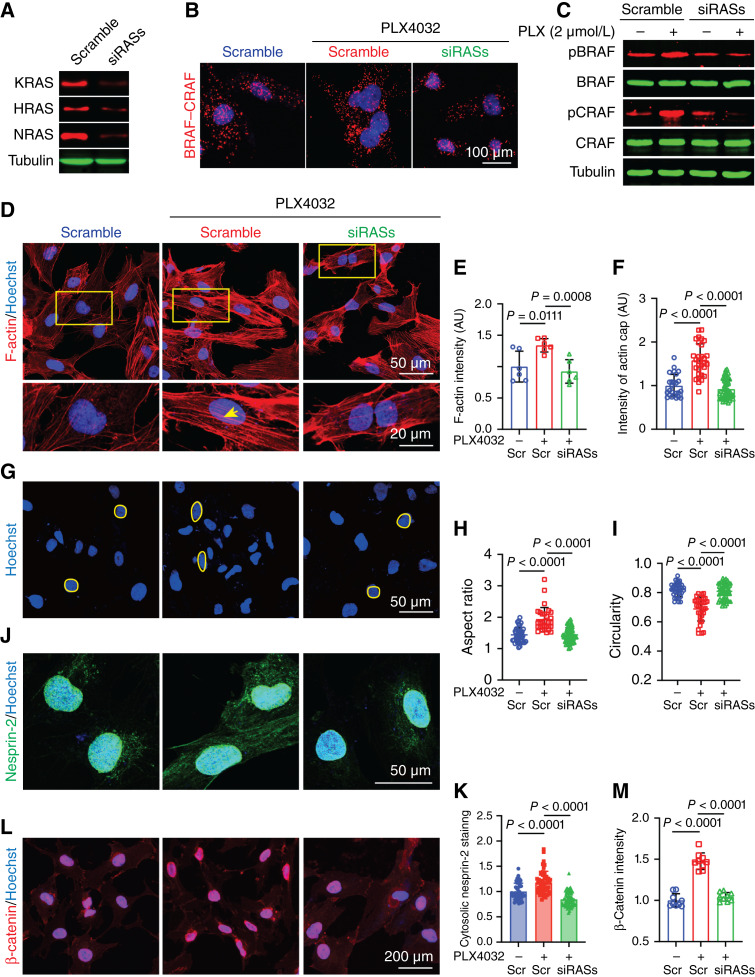
BRAFi-induced RAF activation is RAS-dependent. **A,** Western blot showing effective silencing of KRAS, HRAS, and NRAS expression in iM27 cells using siRNAs. **B,** Representative PLA images showing BRAF and CRAF heterodimerization in iM27 cells transfected with scramble siRNA (Scr), scramble siRNA-transfected iM27 cells treated with PLX4032; and RAS-deficient iM27 cells (siRASs) treated with PLX4032. Red dots indicate BRAF–CRAF dimers. **C,** Western blot showing the phosphorylation of BRAF (Ser445) and CRAF (Ser338) in iM27 cells transfected with scramble siRNA and RAS-deficient iM27 with and without PLX4032 treatment. **D,** Confocal images showing F-actin expression and organization in iM27 cells transfected with scramble siRNA, scramble siRNA-transfected iM27 cells treated with PLX4032 (scramble), and RAS-deficient iM27 cells (siRASs) treated with PLX4032. The insets display enlarged views of representative individual cells (marked by yellow boxes). Yellow arrows indicate actin caps. Scale bars are as indicated. **E** and **F,** Quantification of F-actin intensity (**E**) and actin cap intensity (**F**) using ImageJ. Data are presented as the mean ± SD (*n* = 6 randomly selected 20× fields per group for **E**; *n* = 25–52 nuclei per group for **F**). **G,** Confocal images showing nuclei stained with Hoechst in iM27 cells transfected with scramble siRNA, scramble siRNA-transfected iM27 cells treated with PLX4032, and RAS-deficient iM27 cells (siRASs) treated with PLX4032. Yellow circles indicate the approximate nuclear boundaries of representative cells under the different conditions. Scale bars, 50 μm. **H** and **I,** Scatter dot plots showing nuclear morphologic changes in iM27 cells under three different conditions shown in **G**. Nuclear aspect ratio (**H**) and circularity (**I**) were analyzed and quantified from confocal images using ImageJ. Data are presented as the mean ± SD (*n* = 30–67 nuclei per group). **J,** Confocal images showing nesprin-2 distribution in iM27 cells transfected with scramble siRNA, scramble siRNA-transfected iM27 cells treated with PLX4032, and RAS-deficient iM27 cells (siRASs) treated with PLX4032. The yellow arrow indicates abnormal cytosolic localization of nesprin-2. Scale bars, 50 μm. **K,** Quantification of cytosolic nesprin-2 intensity in iM27 cells under three different conditions shown in **J**. Data are presented as the mean ± SD (*n* = 52–55 cells per group). **L,** Representative fluorescence images showing nuclear β-catenin staining in iM27 cells transfected with scramble siRNA, scramble siRNA-transfected iM27 cells treated with PLX4032, and RAS-deficient iM27 cells (siRASs) treated with PLX4032. Scale bars, 200 μm. **M,** Quantification of nuclear β-catenin intensity in iM27 cells under three different conditions shown in **L**. Data are presented as the mean ± SD (*n* = 9 randomly selected 20× fields per group).

RAFs are mediators between RAS-GTPases and downstream MEK and ERK kinases. In WT RAF cells, RAS binding to the RAS-binding domain (RBD) and cysteine-rich domain (CRD) of RAF is essential for RAF activation because it effectively eliminates autoinhibition, releases the kinase domains, and facilitates RAF dimerization and transactivation. To determine whether RAS binding is required for BRAFi-induced RAF dimerization and transactivation, we depleted all RAS isoforms in CAFs using siRNA (Fig. 5A). PLA revealed that the PLX4032-induced increase in BRAF–CRAF heterodimer formation was markedly reduced in RAS-depleted CAFs compared with scramble siRNA-transfected cells (Fig. 5B; Supplementary Fig. S13). Consistent with this, the phosphorylation of BRAF at Ser445 and CRAF at Ser338 was not elevated following PLX4032 treatment in RAS-depleted cells (Fig. 5C), confirming that RAS is involved in BRAFi-driven RAF dimerization and phosphorylation.

We next examined whether disruption of the RAS–RAF signaling axis affects cytoskeletal remodeling, nuclear morphology, and β-catenin nuclear accumulation. Confocal imaging of F-actin expression and distribution revealed that PLX4032-induced actin polymerization and actin cap formation were suppressed by RAS depletion (Fig. 5D–F; Supplementary Fig. S14A–S14C). Similarly, RAS depletion reversed PLX4032-induced nuclear deformation, with the nuclear aspect ratio returning to ∼1.44 and the circularity increasing to ∼0.81 (Fig. 5G–I; Supplementary Fig. S14D–S14F). In addition, the abnormal cytosolic redistribution of nesprin-2 observed after PLX4032 treatment was reversed in RAS-depleted cells (Fig. 5J and K; Supplementary Fig. S14G and S14H). As a result, the increased nuclear accumulation of β-catenin induced by PLX4032 was significantly diminished in RAS-depleted CAFs, as measured by the average intensity of nuclear β-catenin (Fig. 5L and M; Supplementary Fig. S14I and S14J). These findings support a model in which RAS-dependent release of RAF autoinhibition is required for BRAFi-induced RAF activation, nuclear deformation, and β-catenin accumulation in CAFs.

### ROCK signaling mediates BRAFi- and stiffness-induced cytoskeletal remodeling, nuclear deformation, and β-catenin accumulation in CAFs

**Figure 6.**
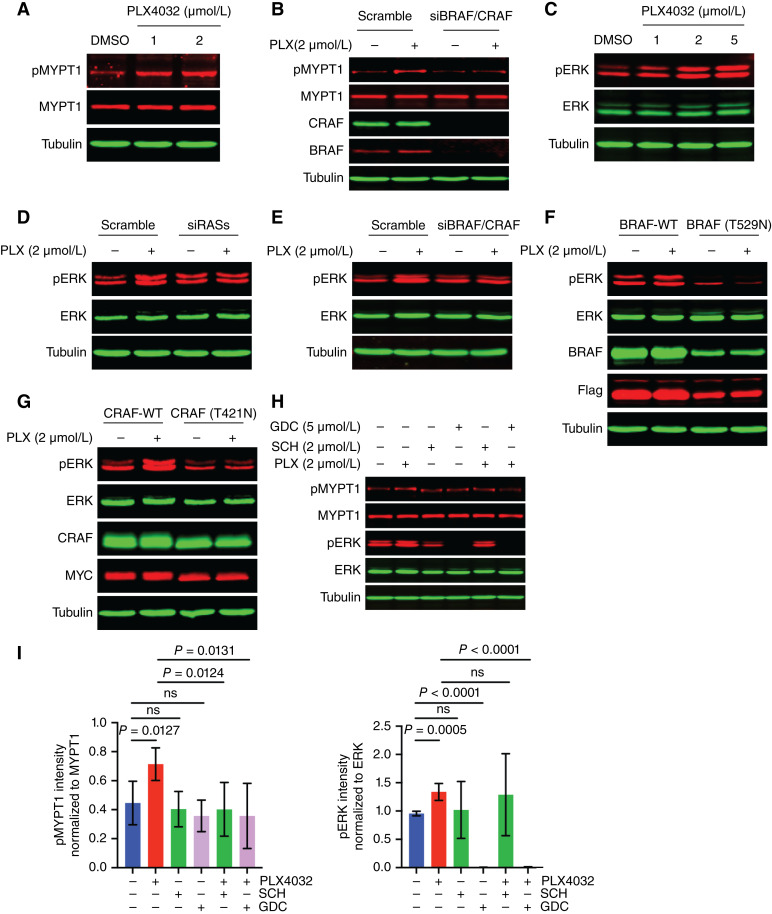
RAS–RAF–ERK signaling contributes to BRAFi-induced ROCK activation. **A,** Western blot showing increased MYPT1 phosphorylation in PLX4032-treated iM27 cells. **B,** Western blot showing MYPT1 phosphorylation in iM27 cells transfected with scramble siRNA and BRAF/CRAF siRNA (siBRAF/CRAF) with or without PLX4032 treatment. **C,** Western blot showing increased ERK phosphorylation in iM27 cells in response to increasing PLX4032 concentrations. **D,** Western blot showing ERK phosphorylation in scramble siRNA-transfected iM27 cells and RAS-deficient iM27 cells (siRASs) with or without PLX4032 treatment. **E,** Western blot showing ERK phosphorylation in iM27 cells transfected with scramble siRNA and BRAF/CRAF siRNA (siBRAF/CRAF) with or without PLX4032 treatment. **F** and **G,** Western blots showing ERK phosphorylation in BRAF-deficient iM27 cells overexpressing WT BRAF and BRAF (T529N; **F**) and in CRAF-deficient iM27 cells overexpressing WT CRAF and CRAF (T421N; **G**) with and without PLX4032 treatment. **H,** Western blot showing MYPT1 and ERK phosphorylation in iM27 cells treated with DMSO, PLX4032, ERK inhibitor SCH772984 (SCH), MEK inhibitor GDC0973 (GDC), or PLX4032 in combination with SCH or GDC. **I,** Quantification of MYPT1 and ERK phosphorylation under the indicated conditions in **H**. Data are presented as the mean ± SD (*n* = 5 independent biological replicates per group). ns, not significant.

To determine how RAF activation promotes actin fiber polymerization, we focused on the ROCK signaling pathway, a key regulator of the actin cytoskeleton. Notably, we observed activation of the ROCK pathway following PLX4032 treatment. MYPT1, a well-known substrate of ROCK that regulates actin contraction and relaxation, was phosphorylated upon PLX4032 exposure (Fig. 6A). Phosphorylation of MYPT1 inhibits its activity, thereby maintaining MLC phosphorylation and promoting actin filament polymerization and contraction (46). Ablation of BRAF and CRAF inhibited PLX4032-induced MYPT1 phosphorylation (Fig. 6B; Supplementary Fig. S15A).

To assess possible molecular interactions between the RAS–RAF signaling pathway and ROCK signaling, we first evaluated the status of ERK, which is an established downstream effector of RAF kinases (47), in response to increasing PLX4032 and GSK2118436 concentrations in two CAF lines. The results showed that BRAFi treatment increased ERK phosphorylation (Fig. 6C; Supplementary Fig. S15B–S15D). This response was diminished upon RAS depletion (Fig. 6D; Supplementary Fig. S15E) and RAF depletion (Fig. 6E; Supplementary Fig. S15F), confirming that RAS–RAF acts upstream of BRAFi-induced ERK activation. Furthermore, PLX4032 did not promote ERK phosphorylation in cells expressing BRAFi-resistant BRAF (T529N) or CRAF (T421N) mutants, indicating that BRAFi-driven RAF activation is required for downstream ERK signaling (Fig. 6F and G). PLX4032-induced MYPT1 phosphorylation was inhibited by cotreatment with the MEK inhibitor GDC0973 and the ERK inhibitor SCH772984 (Fig. 6H–J; Supplementary Fig. S15G), confirming the involvement of MEK and ERK in PLX4032-driven ROCK activation. In addition, GSK-3β phosphorylation at Serine 9 was significantly increased in CAFs following PLX4032 treatment (Supplementary Fig. S15H), indicating that GSK-3β inactivation may contribute to nuclear β-catenin accumulation. Immunostaining for β-catenin also showed that the PLX4032-induced increase in nuclear β-catenin was reduced by ERK inhibition (Supplementary Fig. S15I and S15J).

**Figure 7.**
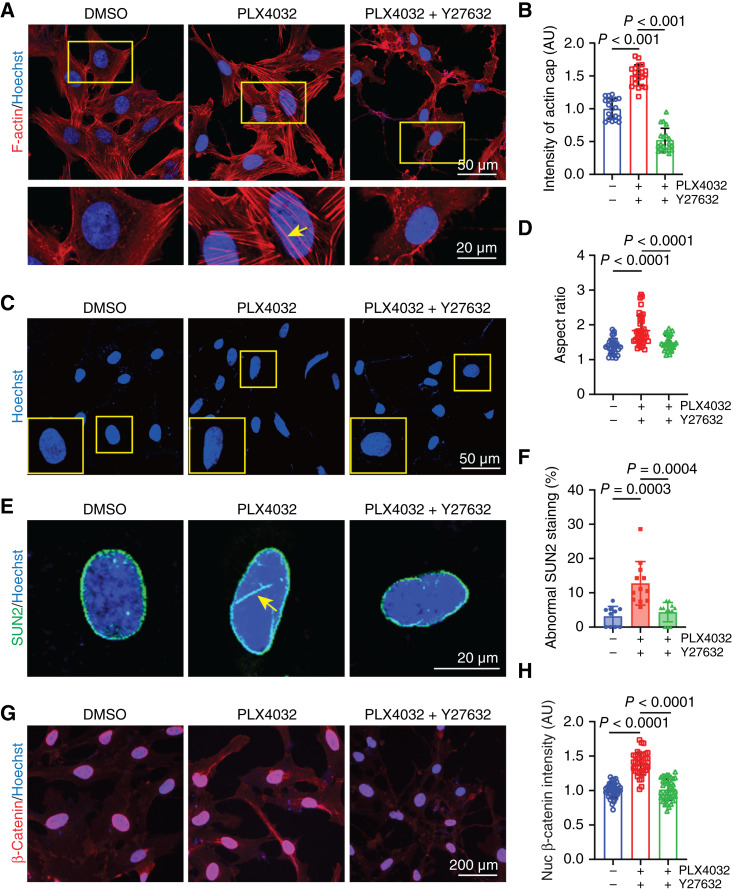
ROCK inhibition reverses BRAFi-induced actin remodeling, nuclear deformation, and β-catenin accumulation in CAFs. **A,** Confocal images showing F-actin expression and organization in iM27 cells treated with DMSO, PLX4032, or the combination of PLX4032 and the ROCK inhibitor Y27632. Insets show enlarged views of representative individual cells highlighted by yellow boxes. Yellow arrows indicate actin caps. Scale bars are as indicated. **B,** Quantification of actin cap intensity from (**A**) using ImageJ. Data are presented as the mean ± SD (*n* = 20 nuclei per group). **C,** Representative confocal images of nuclear morphology visualized by Hoechst staining in iM27 cells treated with DMSO, PLX4032, and the combination of PLX4032 and the ROCK inhibitor Y27632. Yellow circles indicate representative nuclei in each group. Scale bars, 50 μm. **D,** Scatter dot plots showing nuclear morphologic changes in iM27 cells under three different conditions. Nuclear aspect ratio was analyzed and quantified from confocal images from (**C**) using ImageJ. The data are presented as the mean ± SD (*n* = 27–38 nuclei per group). **E,** Representative confocal images showing SUN2 distribution in iM27 cells treated with DMSO, PLX4032, and the combination of PLX4032 and the ROCK inhibitor Y27632. The yellow arrow indicates abnormal nuclear localization of SUN2 in PLX4032-treated cells. Scale bars, 20 μm. **F,** Quantification of the percentage of iM27 cells exhibiting abnormal SUN2 staining under three different conditions from **E**. The data are presented as the mean ± SD (*n* = 10–12 random 40× fields). **G,** Representative fluorescence images showing nuclear β-catenin staining in iM27 cells treated with DMSO, PLX4032, or the combination of PLX4032 and the ROCK inhibitor Y27632. Scale bars, 200 μm. **H,** Quantitative comparison of nuclear β-catenin intensity in iM27 cells under three different conditions from **G**. Data are presented as the mean ± SD (*n* = 40 nuclei per group).

Next, we investigated whether the inhibition of ROCK signaling could reverse the effects of PLX4032 on nuclear deformation and β-catenin nuclear accumulation in CAFs. To this end, CAFs were treated with PLX4032 and a well-established ROCK inhibitor Y27632 (48). Compared with PLX4032 alone, F-actin staining confirmed effective actin cytoskeletal disruption and diminished actin cap formation under combined treatment (Fig. 7A and B). This observation confirms that PLX4032-induced actin cap formation depends on the activation of the ROCK pathway. Furthermore, PLX4032-induced nuclear deformation was reversed by Y27632 treatment, as demonstrated by the restoration of nuclear morphology, with the nuclear aspect ratio returning to ∼1.50 (Fig. 7C and D). The aberrant nuclear distribution of SUN2 triggered by PLX4032 was rescued in CAFs treated with Y27632 (Fig. 7E and F). As expected, increased nuclear accumulation of β-catenin induced by PLX4032 was significantly reduced by ROCK inhibition (Fig. 7G and H). These effects were confirmed using another ROCK inhibitor, HA-1077 (49), and in an additional CAF line (Supplementary Fig. S16).

Because increased ECM stiffness triggered actin polymerization, nuclear deformation, and nuclear β-catenin accumulation in CAFs, we tested whether ROCK inhibition exerted similar suppressive effects on CAFs cultured on stiff substrates. CAFs seeded on a stiff substrate with a stiffness of 50 kPa were treated with Y27632 or HA-1077. Upon ROCK inhibition, confocal imaging revealed a marked loss of cytoskeletal actin fibers and actin cap structures (Supplementary Figs. S17A, S17B, and S18A–S18D). Consequently, nuclear deformation was reversed with the nuclear aspect ratio reduced to ∼1.55 (Supplementary Figs. S17C, S17D, and S18E–S18G). The aberrant nuclear distribution of SUN2 induced by stiffness was reduced in the Y27632-treated CAFs (Supplementary Fig. S17E and S17F). Importantly, the increased nuclear accumulation of β-catenin observed in CAFs cultured on stiff substrates was markedly diminished following Y27632 or HA-1077 treatment (Supplementary Figs. S17G, S17H, and S18H–S18K). Collectively, these findings indicate that the ROCK pathway mediates key cytoskeletal and nuclear responses to BRAFi and stiffness, including actin remodeling, nuclear deformation, and β-catenin nuclear accumulation.

## Discussion

In this study, we identified a shared cytoskeleton-to-nucleus signaling mechanism by which CAFs respond to BRAF inhibition and matrix stiffness. Mechanistically, BRAFi activate the RAF–ROCK axis, inducing actin cytoskeletal remodeling and nuclear deformation in CAFs. These changes are associated with altered NE organization and increased nuclear accumulation of β-catenin, a central mediator of Wnt/β-catenin signaling that regulates transcriptional programs linked to cell fate, tissue remodeling, and cancer progression. Matrix stiffness elicited a similar actin–nuclear remodeling response in CAFs, suggesting convergence between pharmacologic and mechanical inputs. Constitutive β-catenin activation in fibroblasts enhanced actin fiber polymerization, intracellular tension, and CAF-like phenotypes, indicating a potential reciprocal relationship between β-catenin signaling and cytoskeletal remodeling. Consistent with this model, β-catenin activation in fibroblasts promoted CAF-associated matrix remodeling and enhanced melanoma growth *in vivo*. Together with our previous finding that β-catenin depletion in CAFs sensitized BRAF-mutant melanoma cells to BRAFi (7), these results support a functional role for CAF β-catenin signaling in melanoma therapeutic response. However, CAFs represent a heterogeneous population, and whether this ROCK–actin–nuclear β-catenin mechanism operates similarly across CAF subtypes, such as inflammatory or antigen-presenting CAFs, remains unclear and warrants further investigation.

Our findings suggest that BRAF inhibition and matrix stiffness converge on ROCK-dependent cytoskeletal and nuclear remodeling associated with β-catenin accumulation and CAF activation, but these two inputs should not be viewed as independent or mutually exclusive mechanisms. Rather, CAF-intrinsic signaling and ECM stiffness are biologically interdependent: BRAFi-induced intracellular signaling can activate CAFs to remodel and stiffen the ECM, whereas increased ECM stiffness can, in turn, reinforce intracellular mechanotransduction pathways involving ROCK activation, cytoskeletal tension, and nuclear β-catenin accumulation. Thus, the data support a reciprocal feed-forward model in which pharmacologic RAF activation and matrix mechanics amplify one another to sustain CAF activation. However, the magnitude and downstream transcriptional consequences of BRAFi-induced versus stiffness-induced signaling may differ. Future transcriptomic and proteomic studies under matched experimental conditions will be needed to distinguish shared versus stimulus-specific CAF activation programs.

**Figure 8.**
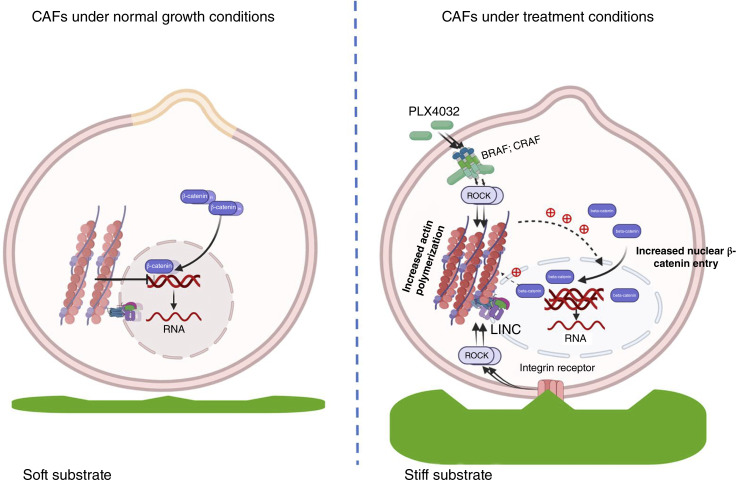
A ROCK–cytoskeleton–β-catenin feed-forward loop reinforces CAF activation. The ROCK–cytoskeleton axis can be activated in CAFs by BRAFi and stiff substrates, leading to nuclear deformation associated with increased β-catenin nuclear accumulation. Nuclear β-catenin further enhances actin stress fiber polymerization, supporting a reciprocal feed-forward loop that continuously reinforces CAF activation and function. [Created in BioRender. Zhang, Y. (2026) https://BioRender.com/kyqkmtp.]

The nucleus, enclosed by a double-layered membrane, tightly regulates protein transport through nuclear pore complexes (NPC; ref. 50). Despite lacking a canonical nuclear localization signal, β-catenin may enter the nucleus via direct NPC interaction or piggyback transport with nuclear proteins such as TCF/LEF (51, 52). In CAFs, our data support a model in which BRAFi- and stiffness-induced ROCK activation promotes cytoskeletal remodeling that deforms the nucleus and alters NE organization (Fig. 8). These changes may influence nuclear pore architecture and transport dynamics, thereby contributing to β-catenin nuclear accumulation, although the precise mechanism remains to be defined. Additionally, BRAFi promoted GSK-3β phosphorylation, which may further stabilize β-catenin and contribute to its nuclear accumulation (53). Although β-catenin accumulation seems to be a key downstream event in this process, we do not exclude the possibility that additional nuclear factors or chromatin remodeling events contribute to CAF activation in parallel. Direct β-catenin perturbation would further clarify its functional contribution to PLX4032-induced CAF phenotypes.

This actin-dependent nuclear deformation is likely mediated through LINC complex remodeling. Transmembrane LINC complexes mechanically connect nuclear lamins and chromatin to the cytoskeleton, thereby maintaining nuclear architecture and transmitting cytoskeletal forces to the nucleus (54). Actin polymerization further promotes actin cap formation, which influences nuclear shape and positioning (37). In both BRAFi-treated CAFs and CAFs cultured on stiff substrates, we observed increased actin cap formation, disorganized SUN2 and nesprin-2 localization, and elongated nuclei. These findings suggest that BRAFi- and stiffness-induced actin remodeling generates mechanical forces that deform the nucleus in association with altered LINC complex organization.

Although BRAFi and matrix stiffness converge on actin polymerization, the upstream stimulatory mechanisms likely differ (Fig. 8). Actin polymerization is regulated in part by the ROCK signaling pathway (55). Although matrix stiffness can activate ROCK signaling through integrin receptors on the cell membrane (56), our findings reveal a distinct mechanism by which BRAFi induces paradoxical RAF activation in CAFs, thereby engaging ROCK signaling. The data indicate that MEK and ERK function downstream of the RAS–RAF axis in CAFs following BRAFi treatment. ERK activation is associated with multiple downstream effects, including ROCK pathway activation and GSK-3β inactivation via phosphorylation. These results support a functional link between the RAF–MEK–ERK axis and ROCK activation, but we cannot exclude the involvement of intermediate signaling nodes or parallel regulatory mechanisms. Further dissection of pathway hierarchy will be necessary to clarify these relationships.

BRAFi were designed to inhibit mutant BRAF carrying the V600E point mutation by binding to the mutant kinase domain and blocking the active site; however, they have been shown to paradoxically activate the MAPK/ERK pathway in cells carrying WT BRAF. Our data suggest that BRAFi treatment promotes BRAF and CRAF dimerization and phosphorylation in BRAF-WT CAFs through RAS-dependent and kinase domain-mediated mechanisms. In addition, BRAFi may stabilize BRAF and CRAF dimers in BRAF-WT CAFs. Increased RAF activity is consistent with BRAFi-induced activation of ERK and ROCK signaling pathways in CAFs, contributing to increased actin polymerization and force transmission to the nucleus. Our investigation also suggests that basal ERK phosphorylation and BRAFi-induced paradoxical RAF activation reflect distinct regulatory contexts. While basal MEK/ERK phosphorylation in CAFs may be partially maintained through RAS-independent or compensatory signaling inputs, RAS activity remains important for BRAFi-induced RAF dimerization and subsequent RAF–MEK–ERK activation.

To explain this context-dependent RAF response, our data support a model in which BRAFi access to the WT RAF kinase domain is required for RAF dimerization and activation, even though BRAFi were designed to fit the mutant BRAF kinase domain and block its abnormal catalytic activity. This differential response may reflect differences in the steric conformation of the kinase domains. As shown in Supplementary Fig. S19, in WT monomeric BRAF, binding of the N-terminal RBD and CRD to the C-terminal kinase domain blocks kinase access to substrates or small-molecule compounds such as BRAFi, thereby maintaining BRAF in an inactive state. However, in the BRAF V600E mutant, this specific substitution within the activation loop of the kinase domain changes its steric conformation and disrupts autoinhibition, leading to constitutive activation even as a monomer without RAS binding. BRAFi can therefore bind the V600E mutant kinase domain and inhibit its kinase activity. In BRAF-WT CAFs, BRAFi access to the kinase domains of BRAF and CRAF seems to occur after RAS binding to the RBD and CRD domains releases autoinhibition. BRAFi then induce conformational changes in the WT kinase domains that promote RAF dimerization and transactivation rather than inhibition.

In summary, our findings reveal that BRAFi reshape CAF behavior through a mechanotransduction mechanism that integrates intracellular signaling with extracellular mechanical cues. Consistent with the reciprocal model described above, BRAFi-induced RAF–ROCK signaling may promote cytoskeletal remodeling, β-catenin activation, and ECM remodeling, whereas altered ECM stiffness may further reinforce CAF activation through ROCK-mediated mechanotransduction. Thus, BRAFi-induced perturbations likely propagate bidirectionally between CAFs and their surrounding microenvironment. These findings position the ROCK–actin–nuclear axis as an important regulator of CAF plasticity and suggest that targeting this pathway could represent a stroma-directed therapeutic strategy in melanoma and other solid tumors.

## Supplementary Material

Supplementary Figure S1 Figure S1. BRAFi induces cytokine/chemokine and ECM gene expression in CAFs

Supplementary Figure S2 Figure S2. PLX4032 induce nuclear deformation in CAFs

Supplementary Figure S3 Figure S3. GSK2118436 induce nuclear deformation in CAFs

Supplementary Figure S4 Figure S4. BRAFi induces nuclear deformation without nuclear rupture in CAFs

Supplementary Figure S5 Figure S5. BRAFi induces actin polymerization and actin cap formation in CAFs

Supplementary Figure S6 Figure S6. Jaspla triggers actin polymerization leading to nuclear deformation

Supplementary Figure S7 Figure S7. BRAFi and ECM stiffness promote β-catenin nuclear accumulation via actin-driven nuclear deformation

Supplementary Figure S8 Figure S8. Nuclear β-catenin drives CAF activation, cytoskeletal remodeling, and contractility in vitro

Supplementary Figure S9 Figure S9. Nuclear β-catenin drives CAF activation, cytoskeletal remodeling, and contractility in vitro.

Supplementary Figure S10 Figure S10. BRAFi binds to the RAF kinase domain and promotes BRAF and CRAF dimerization

Supplementary Figure S11 Figure S11. BRAF and CRAF kinase domains are involved in PLX4032-induced nuclear β-catenin accumulation

Supplementary Figure S12 Figure S12. BRAF and CRAF kinase domains are involved in BRAFi-induced BRAF and CRAF dimerization

Supplementary Figure S13 Figure S13. RAS is involved in PLX4032-induced BRAF and CRAF heterodimerization

Supplementary Figure S14 Figure S14. BRAFi-induced nuclear deformation and β-catenin accumulation are RAS dependent

Supplementary Figure S15 Figure S15. ERK signaling is downstream of BRAFi-induced RAF activation

Supplementary Figure S16 Figure S16. ROCK inhibition reverses PLX4032-induced nuclear deformation, actin polymerization, and β-catenin nuclear accumulation in CAFs

Supplementary Figure S17 Figure S17. ROCK inhibition reverses stiffness-induced actin remodeling, nuclear deformation, and beta-catenin accumulation in CAFs

Supplementary Figure S18 Figure S18. ROCK inhibition reverses stiffness-induced nuclear deformation, actin polymerization, and beta-catenin nuclear accumulation in CAFs

Supplementary Figure S19 Figure S19. BRAFi promotes BRAF and CRAF dimerization and transactivation in CAFs

## Data Availability

The raw data generated in this study are available from the corresponding author upon request.
