# Supplementary material for: BRAF Inhibition–Associated Nuclear Remodeling is Linked to Cancer-Associated Fibroblast Activation
Source: Cancer Res Commun. 2026 Jul 16;6(7):1693–713. doi: 10.1158/2767-9764.CRC-25-0682 (PMC13373777; doi:10.1158/2767-9764.CRC-25-0682)
Supplement: Supplementary Figure S1 — Figure S1. BRAFi induces cytokine/chemokine and ECM gene expression in CAFs [file crc-25-0682_supplementary_figure_s1_suppsf1.docx]

**
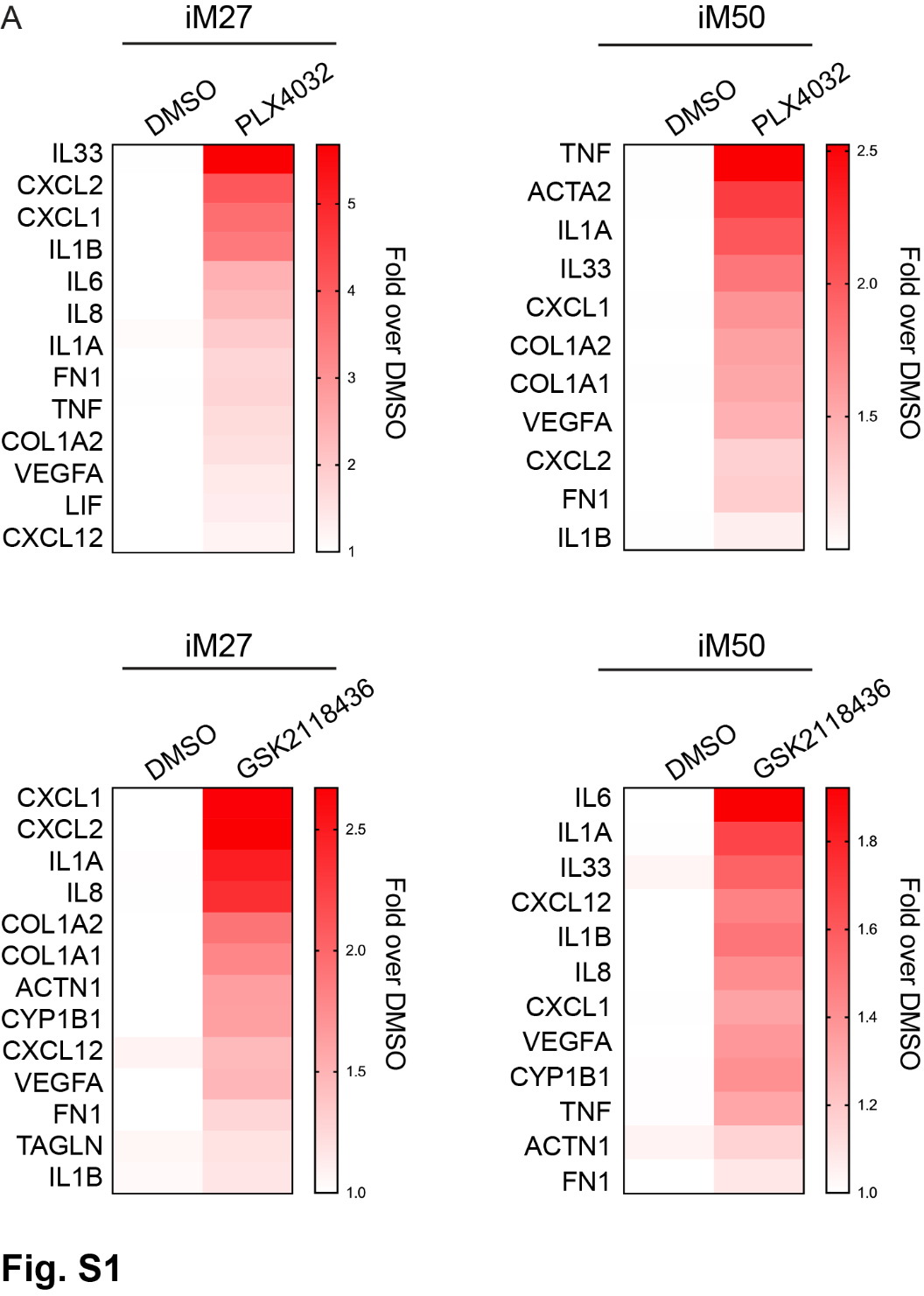
**

**Supplementary Figure S1. BRAFi induces cytokine/chemokine and ECM gene expression in CAFs**

(A) Heatmaps showing qRT-PCR analysis of cytokine/chemokine and ECM-related gene expression in the indicated CAFs treated with DMSO or BRAFis (PLX4032 and GSK2118436). Expression level is shown as fold change relative to DMSO-treated controls.
