# Supplementary material for: BRAF Inhibition–Associated Nuclear Remodeling is Linked to Cancer-Associated Fibroblast Activation
Source: Cancer Res Commun. 2026 Jul 16;6(7):1693–713. doi: 10.1158/2767-9764.CRC-25-0682 (PMC13373777; doi:10.1158/2767-9764.CRC-25-0682)
Supplement: Supplementary Figure S2 — Figure S2. PLX4032 induce nuclear deformation in CAFs [file crc-25-0682_supplementary_figure_s2_suppsf2.docx]

**
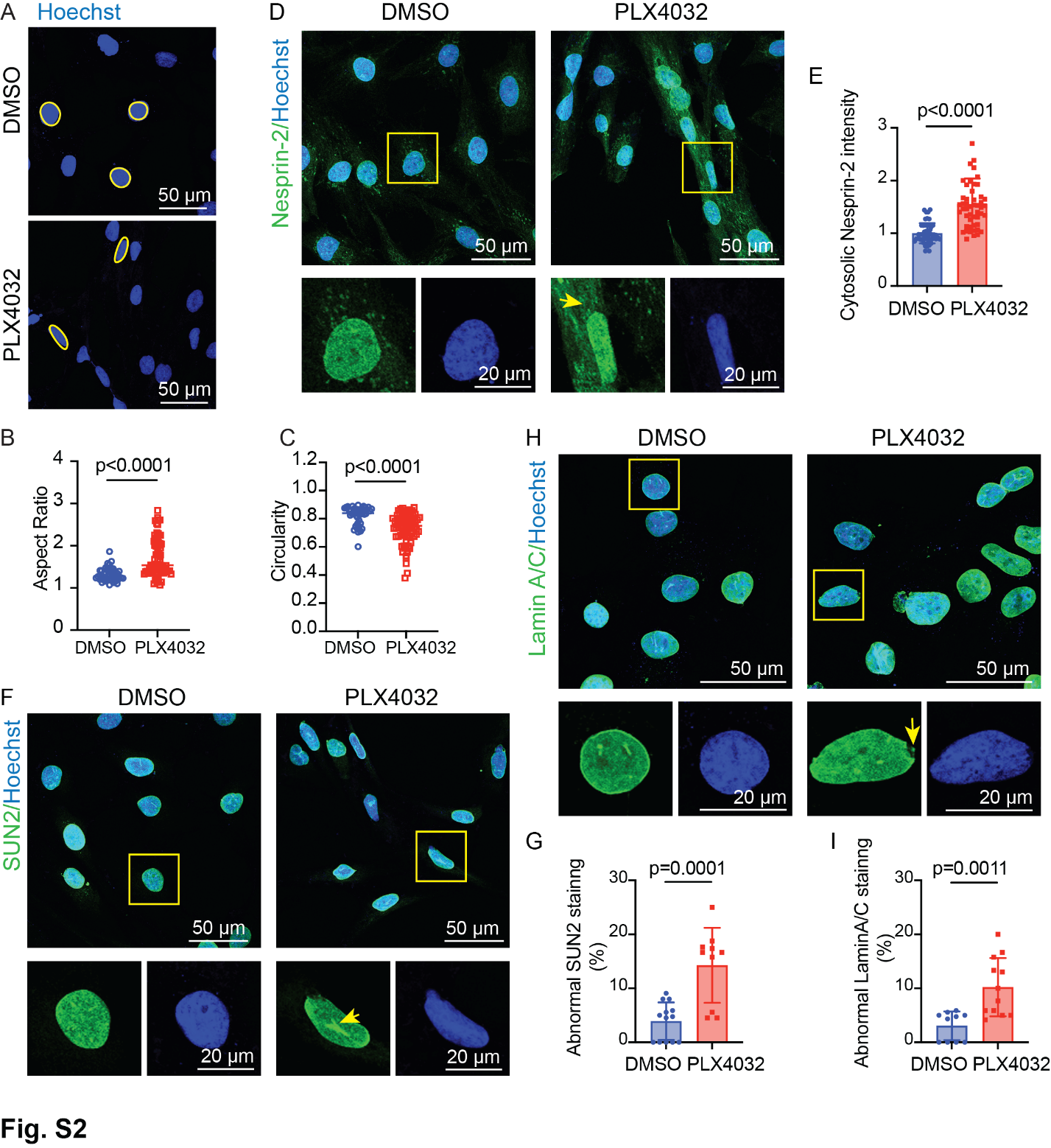
**

**Supplementary Figure S2. PLX4032 induce nuclear deformation in CAFs**

(A) Representative confocal images showing nuclei stained with Hoechst in iM50 cells with or without PLX4032 treatment. Yellow circles indicate the approximate nuclear boundaries of representative cells under the different conditions. Scale bar: 50 μm.

(B, C) Scatter dot plots showing the nuclear morphological parameters in iM50 cells with or without PLX4032 treatment. Nuclear aspect ratio (B) and circularity (C) were analyzed and quantified from confocal images using ImageJ. Data are presented as mean ± SD (n = 44–76 nuclei per group).

(D) Confocal images showing immunostaining of Nesprin-2 in iM50 cells with or without PLX4032 treatment. Small panels show single-channel fluorescence images of representative cells highlighted by yellow boxes in the larger images above. Abnormal distribution patterns of Nesprin-2 in PLX4032-treated iM50 cells is indicated by yellow arrow. Scale bar as indicated.

(E) Quantification of cytosolic Nesprin-2 intensity in iM50 cells under indicated conditions. Data are presented as mean ± SD (n = 49–57 CAFs).

(F) Confocal images showing immunostaining of SUN2 in iM50 cells with or without PLX4032 treatment. Small panels show single-channel fluorescence images of representative cells highlighted by yellow boxes in the larger images above. Disorganized SUN2 distribution pattern in PLX4032-treated iM50 cells is indicated by yellow arrow. Scale bar as indicated.

(G) Quantification of the percentages of iM50 showing abnormal staining of SUN2 under indicated conditions. Data are presented as mean ± SD (n = 10–15 random 40× fields).

(H) Confocal images showing immunostaining of nuclear Lamin A/C in iM50 cells with or without PLX4032 treatment. Small panels show single-channel fluorescence images of representative cells highlighted by yellow boxes in the larger images above. Disorganized Lamin A/C distribution in PLX4032-treated iM50 cells is indicated by yellow arrow. Scale bar as indicated.

(I) Quantification of the percentages of iM50 showing disturbed Lamin A/C nuclear organization under indicated conditions. Data are presented as mean ± SD (n = 10–15 random 40× fields).
