# Supplementary material for: BRAF Inhibition–Associated Nuclear Remodeling is Linked to Cancer-Associated Fibroblast Activation
Source: Cancer Res Commun. 2026 Jul 16;6(7):1693–713. doi: 10.1158/2767-9764.CRC-25-0682 (PMC13373777; doi:10.1158/2767-9764.CRC-25-0682)
Supplement: Supplementary Figure S3 — Figure S3. GSK2118436 induce nuclear deformation in CAFs [file crc-25-0682_supplementary_figure_s3_suppsf3.docx]

**
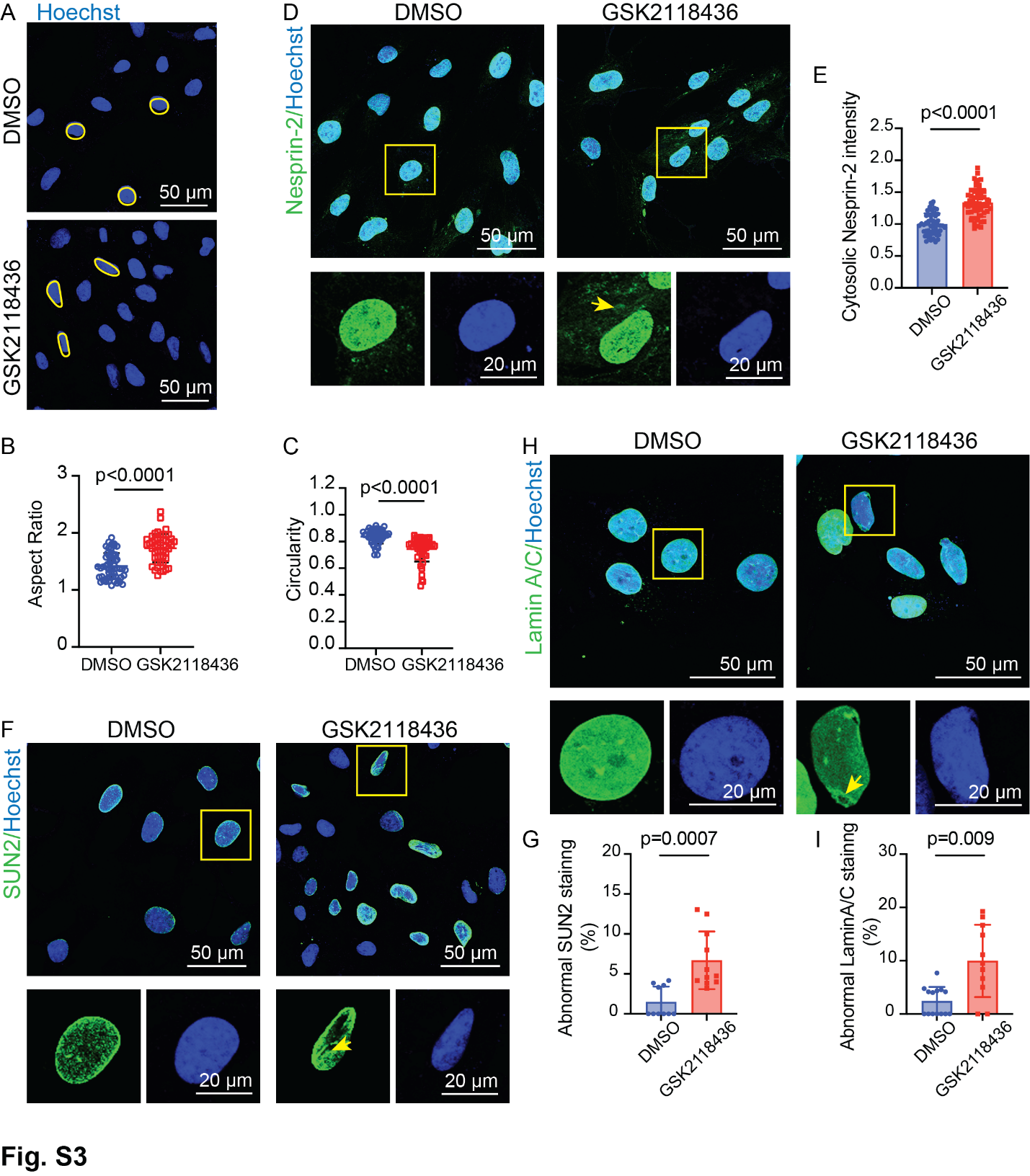
**

**Supplementary Figure S3. GSK2118436 induce nuclear deformation in CAFs**

(A) Representative confocal images showing nuclei stained with Hoechst in iM27 cells with or without GSK2118436 treatment. Yellow circles indicate the approximate nuclear boundaries of representative cells under the different conditions. Scale bar: 50 μm.

(B, C) Scatter dot plots showing the nuclear morphological parameters in iM27 cells with or without GSK2118436 treatment. Nuclear aspect ratio (B) and circularity (C) were measured from confocal images and quantified using ImageJ. Data are presented as mean ± SD (n = 47–56 nuclei per group).

(D) Confocal images showing immunostaining of Nesprin-2 in iM27 cells with or without GSK2118436 treatment. Small panels show single-channel fluorescence images of representative cells highlighted by yellow boxes in the larger images above. Abnormal distribution pattern of Nesprin-2 in GSK2118436-treated iM27 cells is indicated by yellow arrow. Scale bar as indicated.

(E) Quantification of cytosolic Nesprin-2 intensity in iM27 cells under indicated conditions. Data are presented as mean ± SD (n = 49–57 CAFs).

(F) Confocal images showing immunostaining of SUN2 in iM27 cells with or without GSK2118436 treatment. Small panels show single-channel fluorescence images of representative cells highlighted by yellow boxes in the larger images above. Disorganized SUN2 distribution pattern in GSK2118436-treated iM27 cells is indicated by yellow arrow. Scale bar as indicated.

(G) Quantification of the percentages of iM27 showing abnormal staining of SUN2 under indicated conditions. Data are presented as mean ± SD (n = 10–15 random 40× fields).

(H) Confocal images showing immunostaining of nuclear Lamin A/C in iM27 cells with or without GSK2118436 treatment. Small panels show single-channel fluorescence images of representative cells highlighted by yellow boxes in the larger images above. Disorganized Lamin A/C distribution in GSK2118436-treated iM27 cells is indicated by yellow arrow. Scale bar as indicated.

(I) Quantification of the percentages of iM27 showing disturbed Lamin A/C nuclear organization under indicated conditions. Data are presented as mean ± SD (n = 10–15 random 40× fields).
