# Supplementary material for: BRAF Inhibition–Associated Nuclear Remodeling is Linked to Cancer-Associated Fibroblast Activation
Source: Cancer Res Commun. 2026 Jul 16;6(7):1693–713. doi: 10.1158/2767-9764.CRC-25-0682 (PMC13373777; doi:10.1158/2767-9764.CRC-25-0682)
Supplement: Supplementary Figure S4 — Figure S4. BRAFi induces nuclear deformation without nuclear rupture in CAFs [file crc-25-0682_supplementary_figure_s4_suppsf4.docx]

**
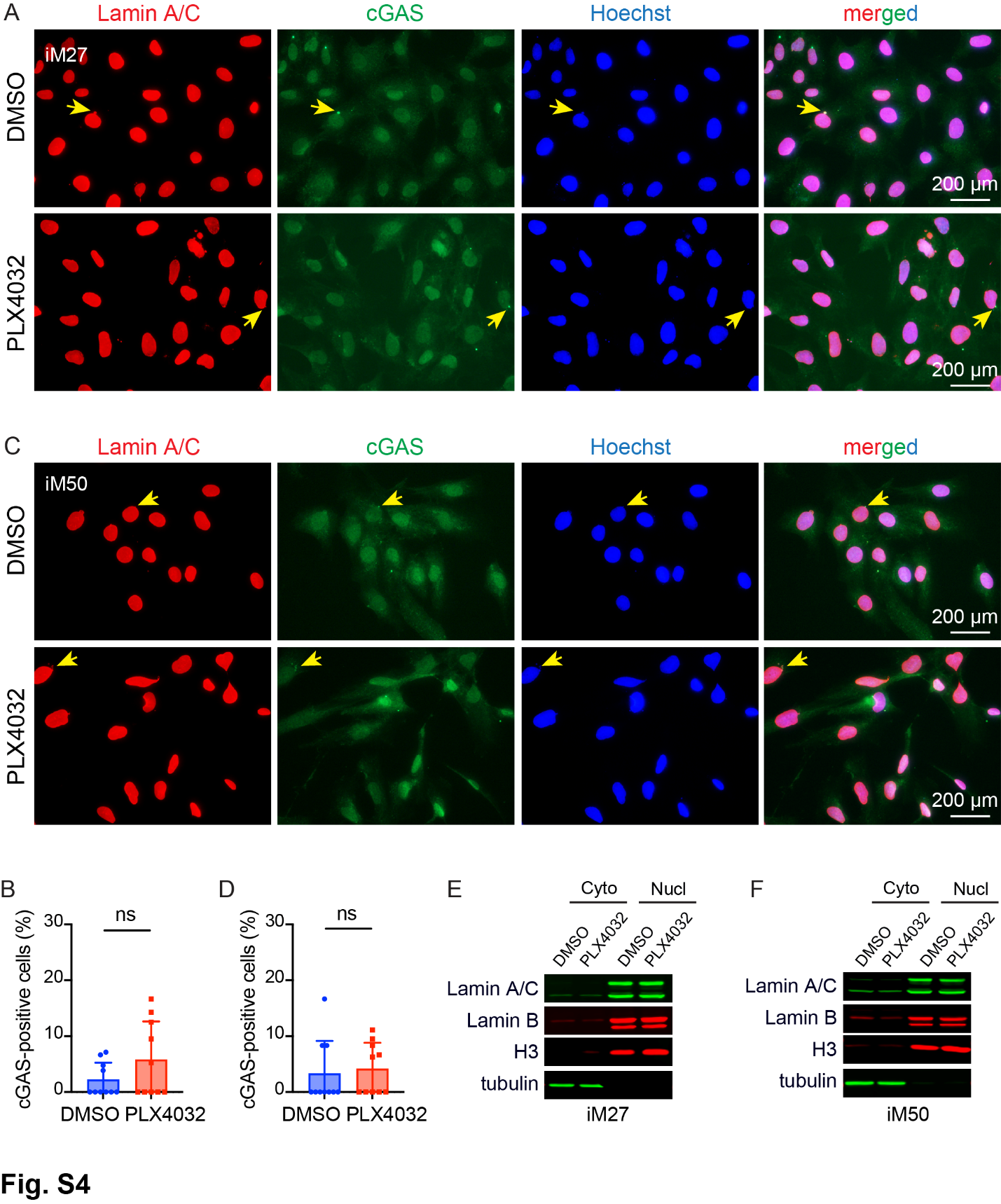
**

**Supplementary Figure S4. BRAFi induces nuclear deformation without nuclear rupture in CAFs**

(A, C) Representative immunofluorescence images showing Lamin A/C and cGAS in iM27 (A) and iM50 (B) cells treated with DMSO or PLX4032. Yellow arrows indicate cGAS accumulation at sites of nuclear rupture. Lamin A/C marks the nuclear envelope. Scale bar: 200 μm.

(B, D) Quantification of cGAS-positive cells, indicating nuclear rupture, in the conditions showed in (A) and (C). Data are presented as mean ± SD (n = 10 random 40× fields)

(E, F) Western blot analysis of Lamin B, Lamin A/C, and histone H3 in cytoplasmic and nuclear fractions isolated from iM27 and iM50 cells treated with DMSO or PLX4032.
