# Supplementary material for: BRAF Inhibition–Associated Nuclear Remodeling is Linked to Cancer-Associated Fibroblast Activation
Source: Cancer Res Commun. 2026 Jul 16;6(7):1693–713. doi: 10.1158/2767-9764.CRC-25-0682 (PMC13373777; doi:10.1158/2767-9764.CRC-25-0682)
Supplement: Supplementary Figure S5 — Figure S5. BRAFi induces actin polymerization and actin cap formation in CAFs [file crc-25-0682_supplementary_figure_s5_suppsf5.docx]

**
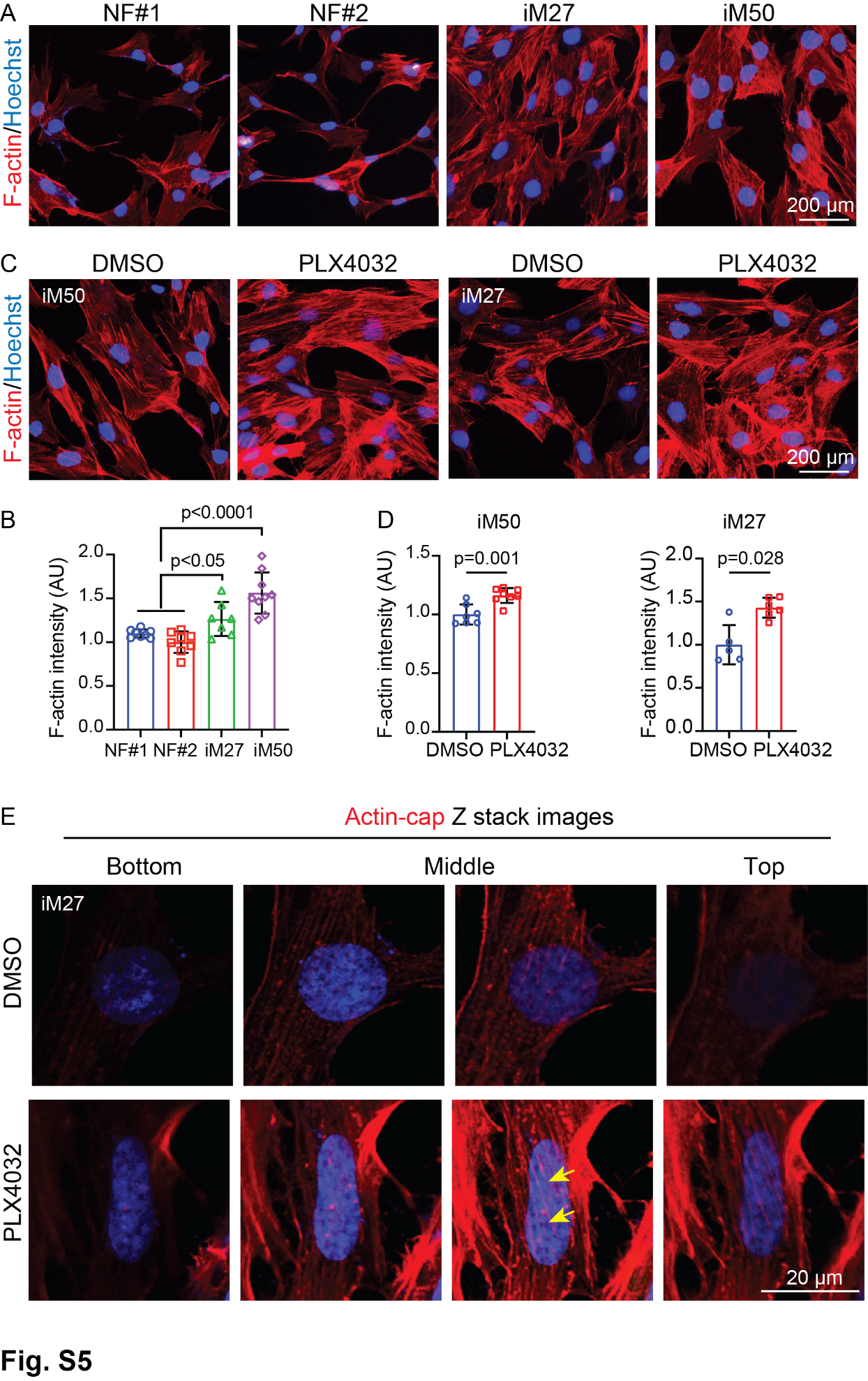
**

**Supplementary Figure S5. BRAFi induces actin polymerization and actin cap formation in CAFs**

(A) Representative fluorescence images showing F-actin expression and organization in normal human fibroblasts (NF#1 and NF#2) and CAFs (iM27 and iM50). Scale bar: 200 μm

(B) Quantification of F-actin intensity in normal human fibroblasts and CAFs. Data are presented as mean ± SD (n = 7–9 random 20× fields).

(C) Representative fluorescence images showing F-actin expression and organization in DMSO-treated and PLX4032-treated iM50 and iM27 CAFs. Scale bar: 200 μm

(D) Quantification of F-actin intensity in DMSO-treated and PLX4032-treated iM50 and iM27 CAFs. Data are presented as mean ± SD (n = 7–8 random 20× fields).

(E) Representative Z-stack confocal images of F-actin in iM27 cells treated with DMSO or PLX4032. A single nucleus was scanned from bottom to top at 1 µm intervals. Actin caps spanning the Hoechst-stained nucleus are indicated by yellow arrows. Scale bar: 200 μm
