# Supplementary material for: BRAF Inhibition–Associated Nuclear Remodeling is Linked to Cancer-Associated Fibroblast Activation
Source: Cancer Res Commun. 2026 Jul 16;6(7):1693–713. doi: 10.1158/2767-9764.CRC-25-0682 (PMC13373777; doi:10.1158/2767-9764.CRC-25-0682)
Supplement: Supplementary Figure S6 — Figure S6. Jaspla triggers actin polymerization leading to nuclear deformation [file crc-25-0682_supplementary_figure_s6_suppsf6.docx]

**
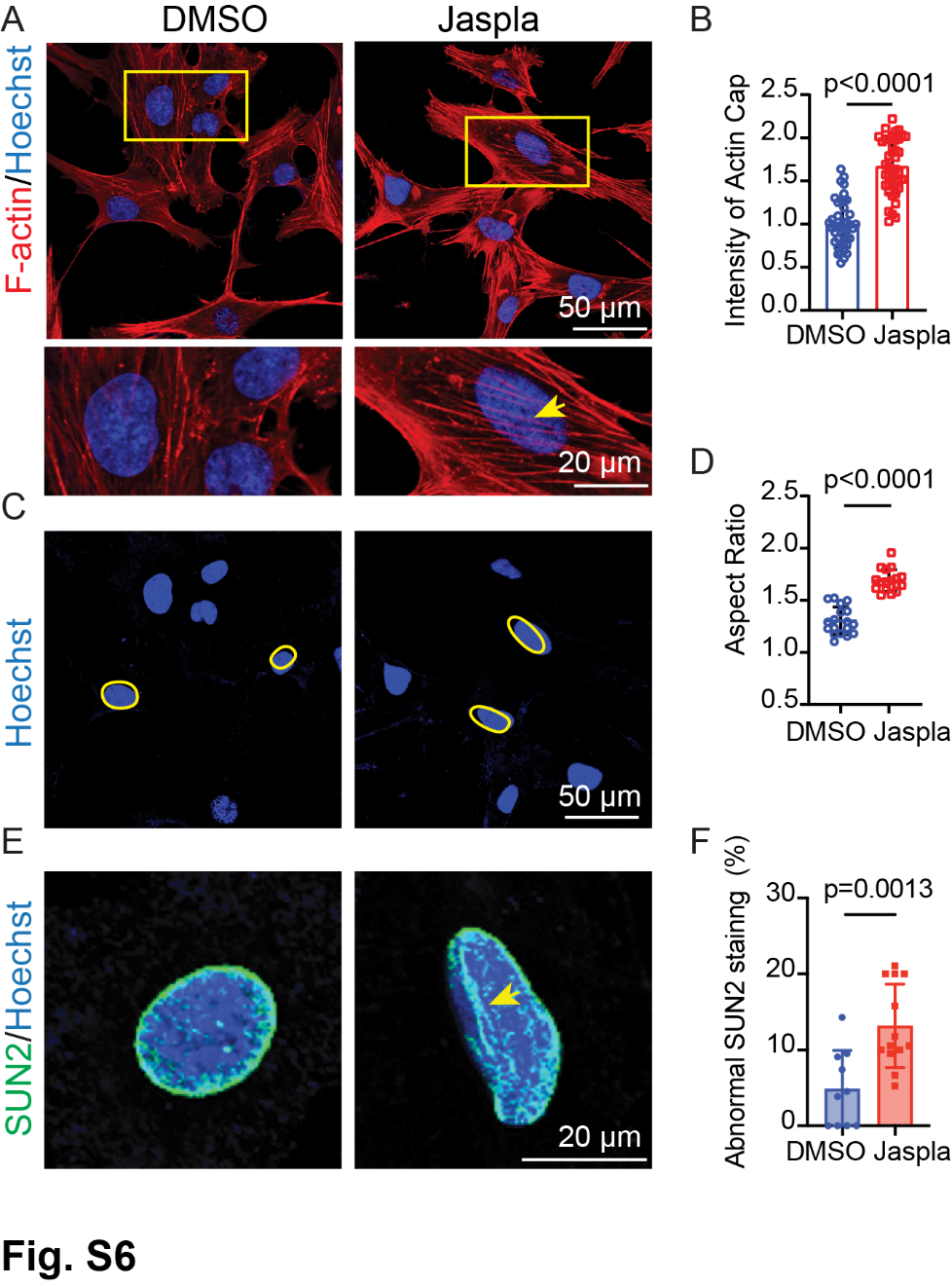
**

**Supplementary Figure S6. Jaspla triggers actin polymerization leading to nuclear deformation**

(A) Confocal images showing F-actin in iM27 cells treated with DMSO or Jaspla. Representative individual cells shown in the insets correspond to the cells highlighted by yellow boxes in the larger images above. Actin caps are indicated by yellow arrows. Scale bars are as indicated.

(B) Quantification of actin cap intensity from (A) using ImageJ. Data are presented as mean ± SD (n = 42 nuclei per group).

(C) Representative confocal images of Hoechst-stained nuclei in iM27 cells treated with DMSO or Jaspla. Yellow circles highlight the approximate nuclear boundaries. Scale bar: 50 μm

(D) Scatter dot plots showing nuclear morphological changes in iM27 ells treated with DMSO or Jaspla. Nuclear aspect ratio was analyzed and quantified from confocal images using ImageJ. Data are presented as mean ± SD (n = 18 nuclei per group).

(E) Confocal images showing SUN2 staining in iM27 cells treated with DMSO or jaspla. Disorganized SUN2 distribution in jaspla-treated iM27 cells is indicated by yellow arrows. Scale bar: 20 μm

(F) Quantification of the percentages of iM27 cells exhibiting abnormal SUN2 staining. Data are presented as mean ± SD (n = 10–13 random 40× fields).
