# Supplementary material for: BRAF Inhibition–Associated Nuclear Remodeling is Linked to Cancer-Associated Fibroblast Activation
Source: Cancer Res Commun. 2026 Jul 16;6(7):1693–713. doi: 10.1158/2767-9764.CRC-25-0682 (PMC13373777; doi:10.1158/2767-9764.CRC-25-0682)
Supplement: Supplementary Figure S7 — Figure S7. BRAFi and ECM stiffness promote β-catenin nuclear accumulation via actin-driven nuclear deformation [file crc-25-0682_supplementary_figure_s7_suppsf7.docx]

**
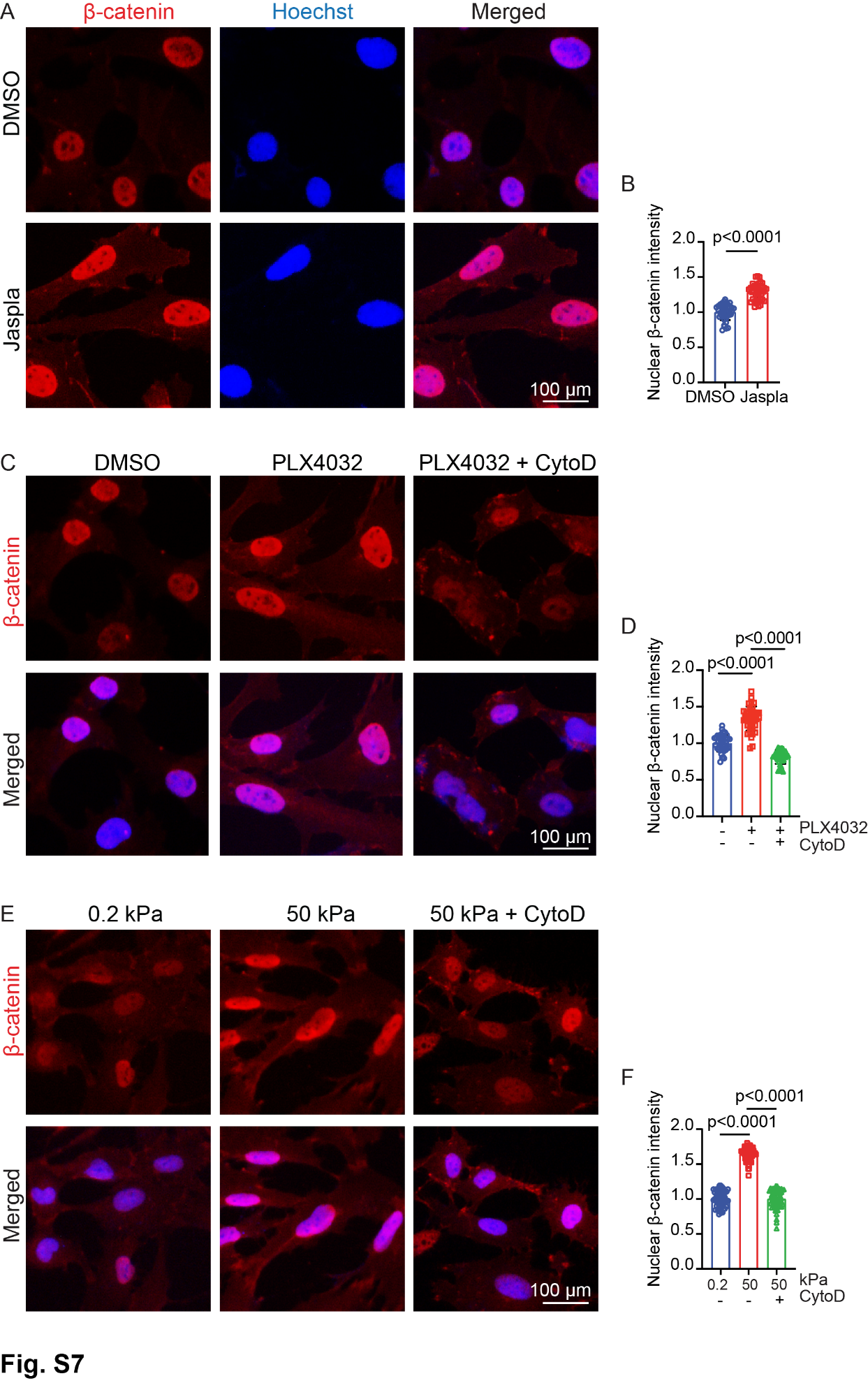
**

**Supplementary Figure S7. BRAFi and ECM stiffness promote β-catenin nuclear accumulation via actin-driven nuclear deformation**

(A) Fluorescence microscopy images showing nuclear β-catenin staining in iM27 cells treated with DMSO or jaspla. Both single channel β-catenin staining and merged images with Hoechst nuclear counterstaining are shown. Scale bar: 100 μm.

(B) Quantitative comparison of nuclear β-catenin intensity in iM27 cells treated with DMSO or Jaspla. Data are presented as mean ± SD (n = 40 cells per group).

(C) Fluorescence microscopy images showing nuclear β-catenin staining in iM27 cells treated with DMSO, PLX4032 or the combination of PLX4032 and CytoD. Both single channel β-catenin staining and merged images with Hoechst nuclear counterstaining are shown. Scale bar: 100 μm.

(D) Quantitative comparison of nuclear β-catenin intensity in iM27 cells treated with DMSO, PLX4032 or the combination of PLX4032 and CytoD. Data are presented as mean ± SD (n = 40 cells per group).

(E) Fluorescence microscopy images showing nuclear β-catenin staining in iM27 cells cultured on soft slides with a stiffness of 0.2 kPa (left), hard slides with a stiffness of 50 kPa (middle), and hard slides with a stiffness of 50 kPa and CytoD treatment (right). Both single channel β-catenin staining and merged images with Hoechst nuclear counterstaining are shown. Scale bar: 100 μm.

(F) Quantitative comparison of nuclear β-catenin intensity in iM27 cells under three different conditions. Data are presented as mean ± SD (n = 60 cells per group).
