# Supplementary material for: BRAF Inhibition–Associated Nuclear Remodeling is Linked to Cancer-Associated Fibroblast Activation
Source: Cancer Res Commun. 2026 Jul 16;6(7):1693–713. doi: 10.1158/2767-9764.CRC-25-0682 (PMC13373777; doi:10.1158/2767-9764.CRC-25-0682)
Supplement: Supplementary Figure S8 — Figure S8. Nuclear β-catenin drives CAF activation, cytoskeletal remodeling, and contractility in vitro [file crc-25-0682_supplementary_figure_s8_suppsf8.docx]

**
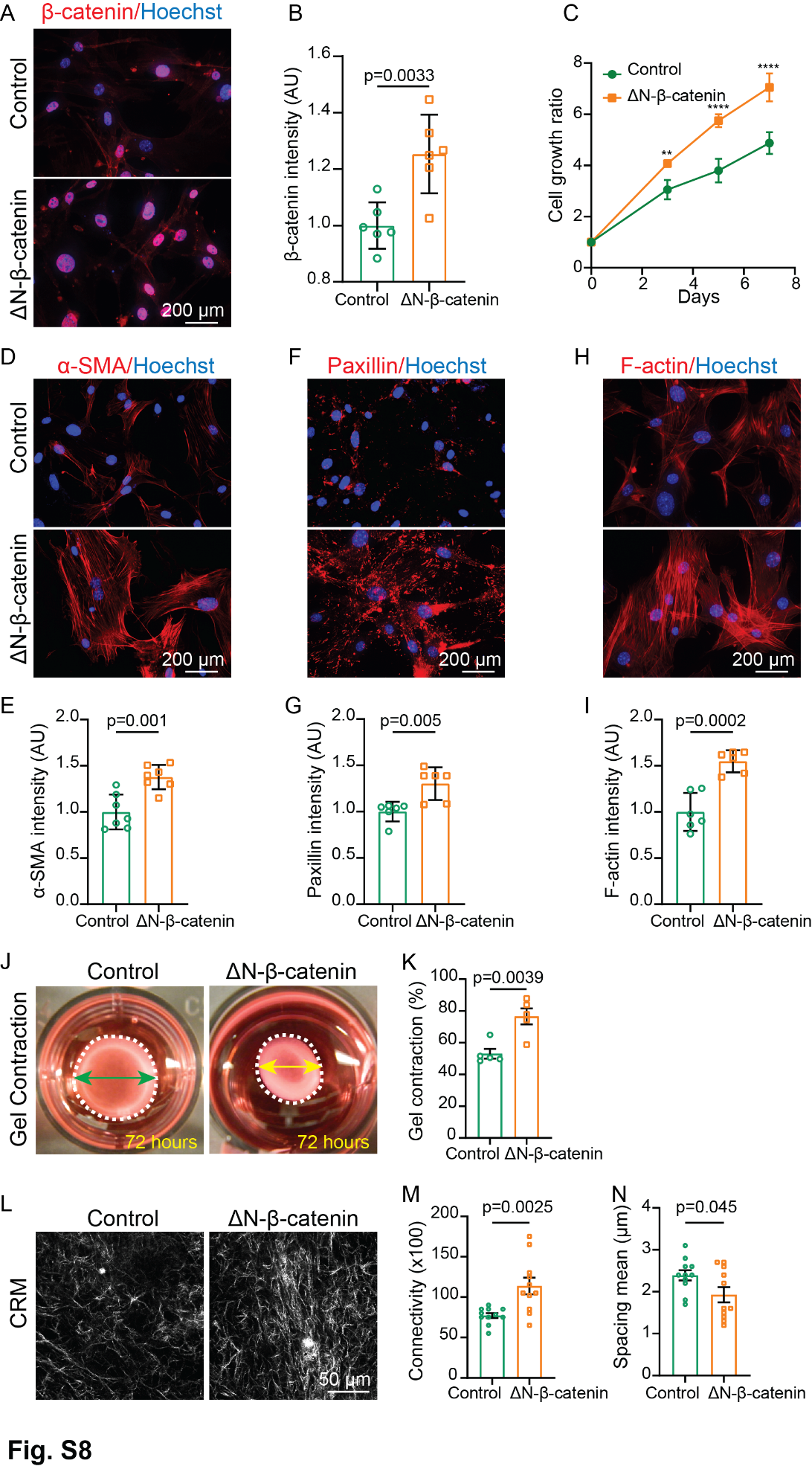
**

**Supplementary Figure S8. Nuclear β-catenin drives CAF activation, cytoskeletal remodeling, and contractility *in vitro***

(A) Representative immunofluorescence images showing β-catenin expression in control fibroblasts (*Col1α2-CreER; Rosa-rtTA*) and β-catenin-overexpressing fibroblasts (*Col1α2-CreER; Rosa-rtTA; TetO-ΔN-β-catenin*). Nuclei were counterstained with Hoechst (blue). Scale bar: 200 μm.

(B) Quantification of nuclear β-catenin levels in control and ΔN-β-catenin-overexpressing fibroblasts. Data are presented as mean ± SD (n = 6 randomly selected 40× fields per group).

(C) Comparison of cell proliferation between control and ΔN-β-catenin-overexpressing fibroblasts. Data are presented as mean ± SD (n = 4 replicates per group).

(D) Representative immunofluorescence images showing α-SMA expression in control and ΔN-β-catenin-overexpressing fibroblasts. Nuclei were counterstained with Hoechst. Scale bar: 200 μm.

(E) Quantification of α-SMA expression in control and ΔN-β-catenin-overexpressing fibroblasts. Data are presented as mean ± SD (n = 6–7 randomly selected 40× fields per group).

(F) Representative immunofluorescence images showing paxillin expression in control and ΔN-β-catenin-overexpressing fibroblasts. Nuclei were counterstained with Hoechst. Scale bar: 200 μm.

(G) Quantification of paxillin expression levels in control and ΔN-β-catenin-overexpressing fibroblasts. Data are presented as mean ± SD (n = 6–7 randomly selected 40× fields per group).

(H) Representative immunofluorescence images showing F-actin expression in control and ΔN-β-catenin-overexpressing fibroblasts. Nuclei were counterstained with Hoechst. Scale bar: 200 μm.

(I) Quantification of F-actin levels in control and ΔN-β-catenin-overexpressing fibroblasts. Data are presented as mean ± SD (n = 6–7 randomly selected 40× fields per group).

(J) Representative images of collagen gel contraction assays for control and ΔN-β-catenin-overexpressing fibroblasts. The gel in each well is circled by a white dashed line. Green (control) and yellow (ΔN-β-catenin) arrows indicate the diameter of the contracted gels after 72 hours.

(K) Quantification of gel contraction (% of initial area) by control and ΔN-β-catenin-overexpressing fibroblasts after 72 hours. Data are presented as mean ± SD (n = 5 replicates per group).

(L) Representative CRM images of collagen gels embedded with control or ΔN-β-catenin-overexpressing fibroblasts after 72 hours. Scale bar: 50 μm

(M, N) CRM images were analyzed using ImageJ with the BoneJ plugin to compare collagen fiber network connectivity and spacing in gels embedded with control and ΔN-β-catenin-overexpressing fibroblasts at 72 hours (n = 11 randomly selected 40× fields per group).
