# Supplementary material for: BRAF Inhibition–Associated Nuclear Remodeling is Linked to Cancer-Associated Fibroblast Activation
Source: Cancer Res Commun. 2026 Jul 16;6(7):1693–713. doi: 10.1158/2767-9764.CRC-25-0682 (PMC13373777; doi:10.1158/2767-9764.CRC-25-0682)
Supplement: Supplementary Figure S9 — Figure S9. Nuclear β-catenin drives CAF activation, cytoskeletal remodeling, and contractility in vitro. [file crc-25-0682_supplementary_figure_s9_suppsf9.docx]

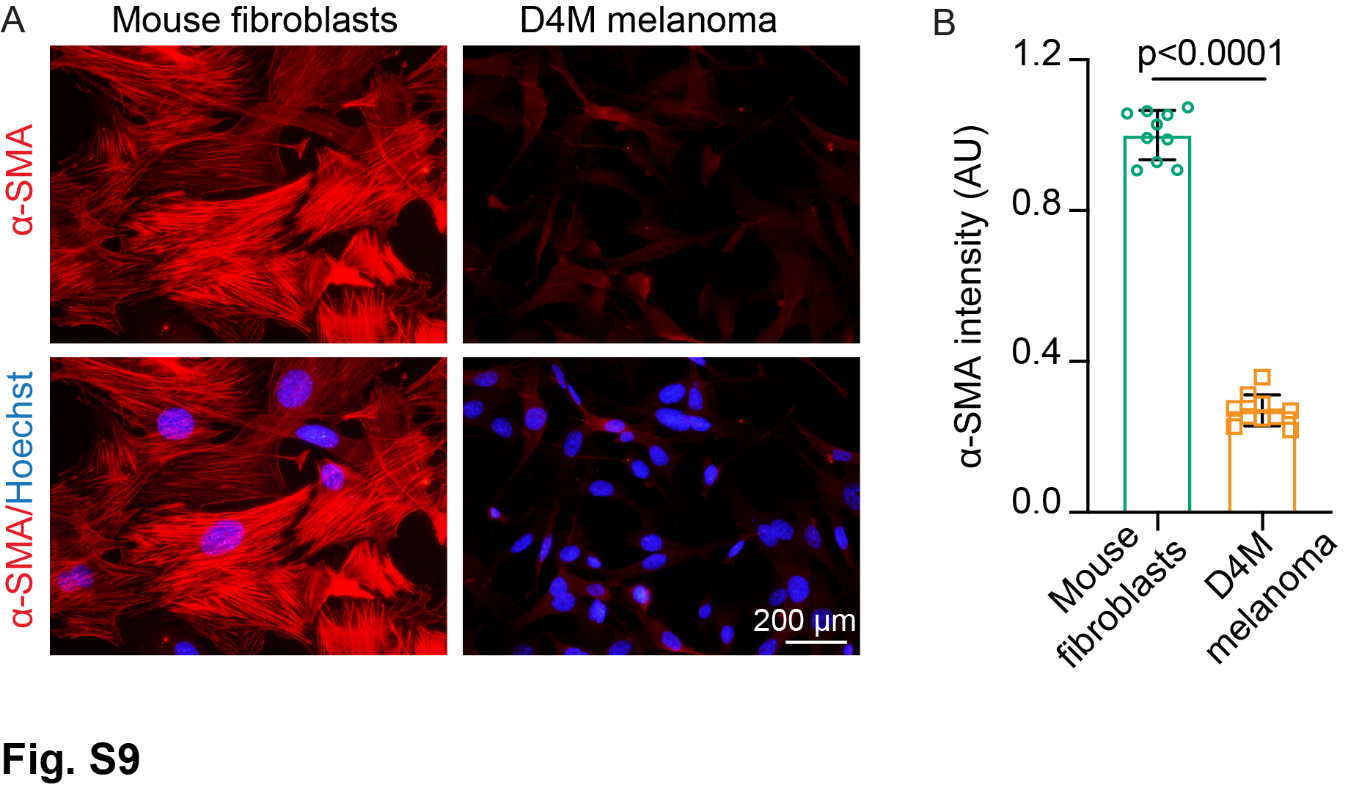


**Supplementary Figure S9. D4M mouse melanoma cells lack α-SMA expression compared with mouse fibroblasts**

(A) Representative immunofluorescence images showing that α-SMA is expressed in mouse fibroblasts but not in D4M melanoma cells. Nuclei were counterstained with Hoechst. Scale bar: 200 μm.

(B) Quantification of α-SMA expression in mouse fibroblasts and D4M melanoma cells. Data are presented as mean ± SD (n = 10 randomly selected 40× fields per group).
