# Supplementary material for: BRAF Inhibition–Associated Nuclear Remodeling is Linked to Cancer-Associated Fibroblast Activation
Source: Cancer Res Commun. 2026 Jul 16;6(7):1693–713. doi: 10.1158/2767-9764.CRC-25-0682 (PMC13373777; doi:10.1158/2767-9764.CRC-25-0682)
Supplement: Supplementary Figure S10 — Figure S10. BRAFi binds to the RAF kinase domain and promotes BRAF and CRAF dimerization [file crc-25-0682_supplementary_figure_s10_suppsf10.docx]

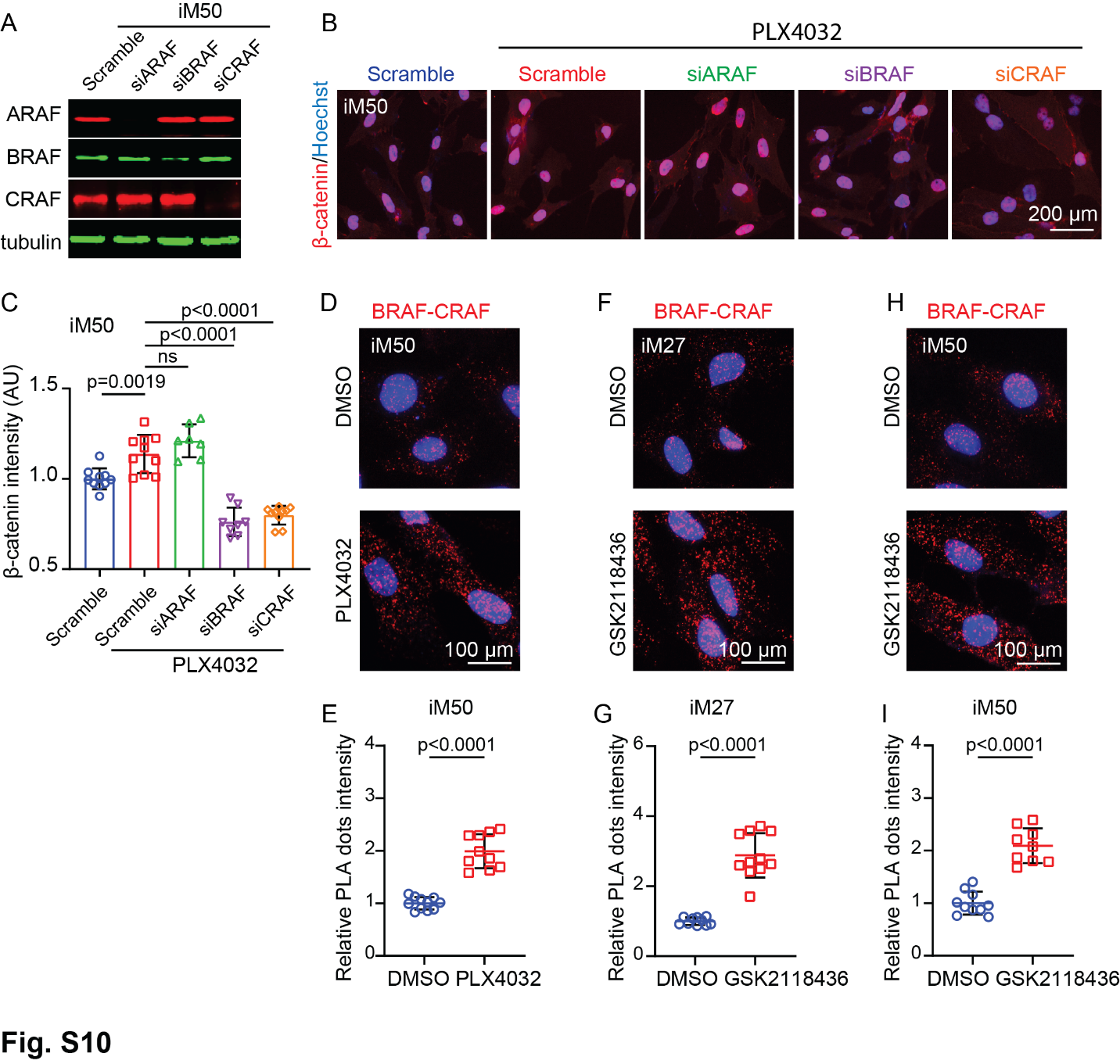


**Supplementary Figure S10. BRAFi binds to the RAF kinase domain and promotes BRAF and CRAF dimerization**

(A) Western blot confirming effective silencing of ARAF, BRAF, or CRAF expression in iM50 cells using the corresponding siRNAs.

(B) Representative fluorescence images showing nuclear β-catenin expression in iM50 cells transfected with scramble siRNA or siRNAs that deplete ARAF, BRAF, or CRAF expression under PLX4032 treatment. iM50 cells transfected with scramble siRNA without PLX4032 treatment were used as the control. Scale bar: 200 μm

(C) Quantification of nuclear β-catenin intensity in iM50 cells and genetically modified iM50 cells as shown in (B) with or without PLX4032 treatment using ImageJ. n = 8 randomly selected 20× fields per group.

(D, F, H) Representative PLA images showing BRAF-CRAF heterodimerization in iM50 cells treated with DMSO or PLX4032 (D), in iM27 cells treated with DMSO or GSK2118436 (F), and in iM50 cells treated with DMSO or GSK2118436 (H). Red dots indicate BRAF-CRAF dimers. Scale bar: 100 μm

(E, G, I) Quantification of PLA signals corresponding to panels (D), (F), and (H), respectively. Data are presented as mean ± SD (n = 9–11 randomly selected 40× fields per group).
