# Supplementary material for: BRAF Inhibition–Associated Nuclear Remodeling is Linked to Cancer-Associated Fibroblast Activation
Source: Cancer Res Commun. 2026 Jul 16;6(7):1693–713. doi: 10.1158/2767-9764.CRC-25-0682 (PMC13373777; doi:10.1158/2767-9764.CRC-25-0682)
Supplement: Supplementary Figure S11 — Figure S11. BRAF and CRAF kinase domains are involved in PLX4032-induced nuclear β-catenin accumulation [file crc-25-0682_supplementary_figure_s11_suppsf11.docx]

**
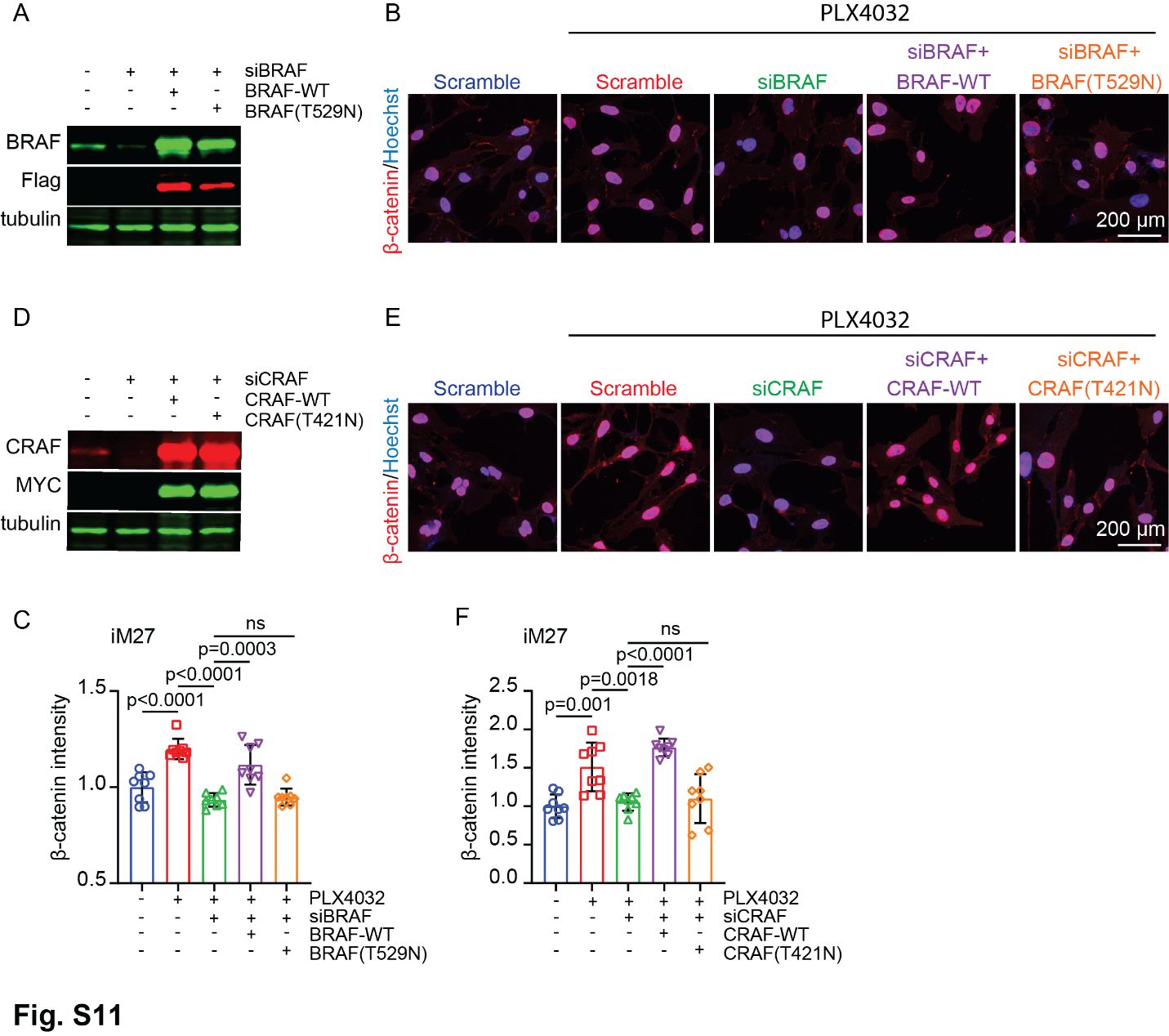
**

**Supplementary Figure S11. BRAF and CRAF kinase domains are involved in PLX4032-induced nuclear β-catenin accumulation**

(A) Western blot showing BRAF expression in iM27 cells transfected with scramble siRNA, iM27 cells transfected with BRAF siRNA (BRAF-deficient iM27), BRAF-deficient iM27 cells overexpressing wild-type BRAF (siBRAF + BRAF-WT), and BRAF-deficient iM27 cells overexpressing BRAF (T529N) (siBRAF + BRAF (T529N)).

(B) Representative fluorescence images showing nuclear β-catenin expression in iM27 cells and genetically modified iM27 cells shown in (A) with or without PLX4032 treatment as indicated. Scale bar: 200 μm.

(C) Quantification of nuclear β-catenin fluorescence intensity in iM27 cells and genetically modified iM27 cells shown in (B). n = 8 randomly selected 20× fields per group.

(D) Western blot showing CRAF expression in iM27 cells transfected with scramble siRNA, iM27 cells transfected with CRAF siRNA (CRAF-deficient iM27), CRAF-deficient iM27 cells overexpressing wild-type CRAF (siCRAF + CRAF-WT), and CRAF-deficient iM27 cells overexpressing CRAF (T421N) (siCRAF + CRAF (T421N)).

(E) Representative fluorescence images showing nuclear β-catenin expression in iM27 cells and genetically modified iM27 cells shown in (D) with or without PLX4032 treatment as indicated. Scale bar: 200 μm.

(F) Quantification of nuclear β-catenin fluorescence intensity in iM27 cells and genetically modified iM27 cells shown in (E). n = 8 randomly selected 20× fields per group.
