# Supplementary material for: BRAF Inhibition–Associated Nuclear Remodeling is Linked to Cancer-Associated Fibroblast Activation
Source: Cancer Res Commun. 2026 Jul 16;6(7):1693–713. doi: 10.1158/2767-9764.CRC-25-0682 (PMC13373777; doi:10.1158/2767-9764.CRC-25-0682)
Supplement: Supplementary Figure S12 — Figure S12. BRAF and CRAF kinase domains are involved in BRAFi-induced BRAF and CRAF dimerization [file crc-25-0682_supplementary_figure_s12_suppsf12.docx]

**
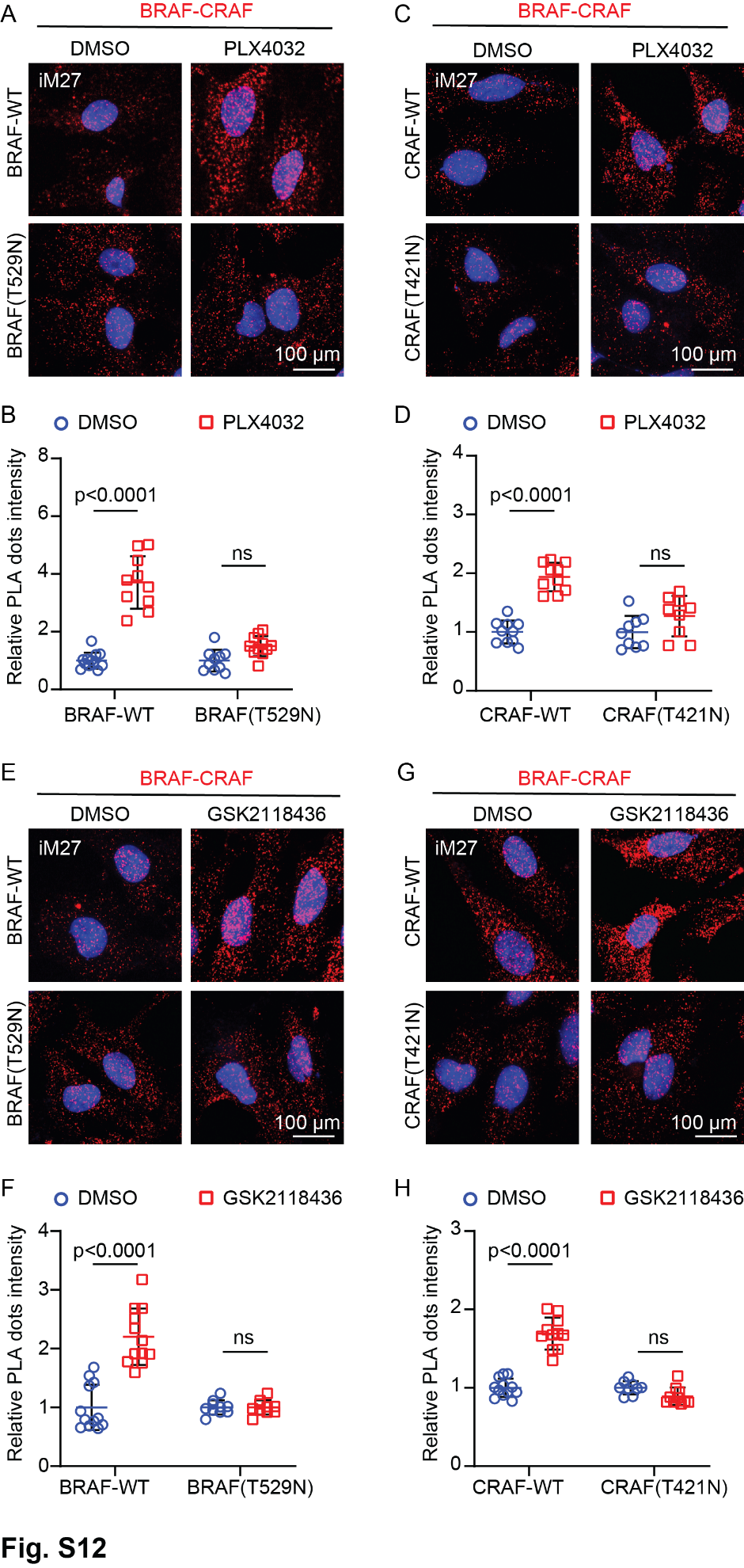
**

**Supplementary Figure S12. BRAF and CRAF kinase domains are involved in BRAFi-induced BRAF and CRAF dimerization**

(A, C) Representative PLA images showing BRAF-CRAF heterodimerization in BRAF-deficient iM27 cells expressing wild-type BRAF or BRAF (T529N) (A) and in CRAF-deficient iM27 cells expressing wild-type CRAF or CRAF (T421N) (C). Cells were treated with PLX4032 and compared with DMSO-treated controls. Red dots correspond to BRAF-CRAF interactions.

(B, D) Quantification of PLA signals (red dots) corresponding to panels (A) and (C). data are presented as mean ± SD (n = 9–12 randomly selected 40× fields per group).

(E, G) Representative PLA images showing BRAF-CRAF heterodimerization in BRAF-deficient iM27 cells expressing wild-type BRAF or BRAF (T529N) (E), and in CRAF-deficient iM27 cells expressing wild-type CRAF or CRAF (T421N) (G). Cells were treated with GSK2118436 and compared with DMSO-treated controls. Red dots indicate BRAF-CRAF dimers.

(F, H) Quantification of PLA signals corresponding to panels (E) and (G). Data are presented as mean ± SD (n = 9–12 randomly selected 40× fields per group).

Scale bar: 100 μm for all images.
