# Supplementary material for: BRAF Inhibition–Associated Nuclear Remodeling is Linked to Cancer-Associated Fibroblast Activation
Source: Cancer Res Commun. 2026 Jul 16;6(7):1693–713. doi: 10.1158/2767-9764.CRC-25-0682 (PMC13373777; doi:10.1158/2767-9764.CRC-25-0682)
Supplement: Supplementary Figure S13 — Figure S13. RAS is involved in PLX4032-induced BRAF and CRAF heterodimerization [file crc-25-0682_supplementary_figure_s13_suppsf13.docx]

**
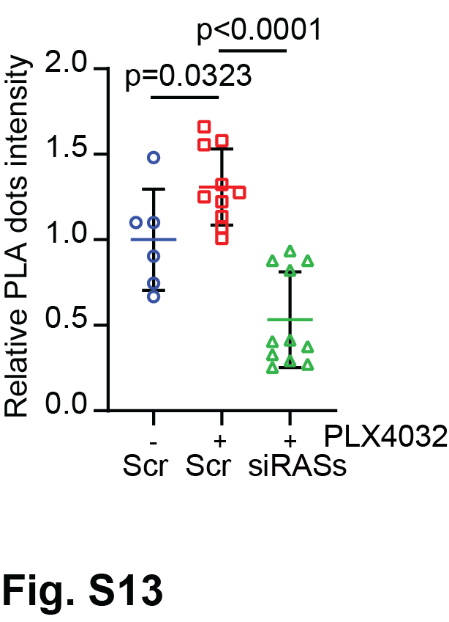
**

**Supplementary Figure S13. RAS is involved in PLX4032-induced BRAF and CRAF heterodimerization**

(A) Quantification of PLA signals (red dots) shown in Fig. 5B. Data are presented as mean ± SD (n = 6–11 randomly selected 40× fields per group).
