# Supplementary material for: BRAF Inhibition–Associated Nuclear Remodeling is Linked to Cancer-Associated Fibroblast Activation
Source: Cancer Res Commun. 2026 Jul 16;6(7):1693–713. doi: 10.1158/2767-9764.CRC-25-0682 (PMC13373777; doi:10.1158/2767-9764.CRC-25-0682)
Supplement: Supplementary Figure S14 — Figure S14. BRAFi-induced nuclear deformation and β-catenin accumulation are RAS dependent [file crc-25-0682_supplementary_figure_s14_suppsf14.docx]

**
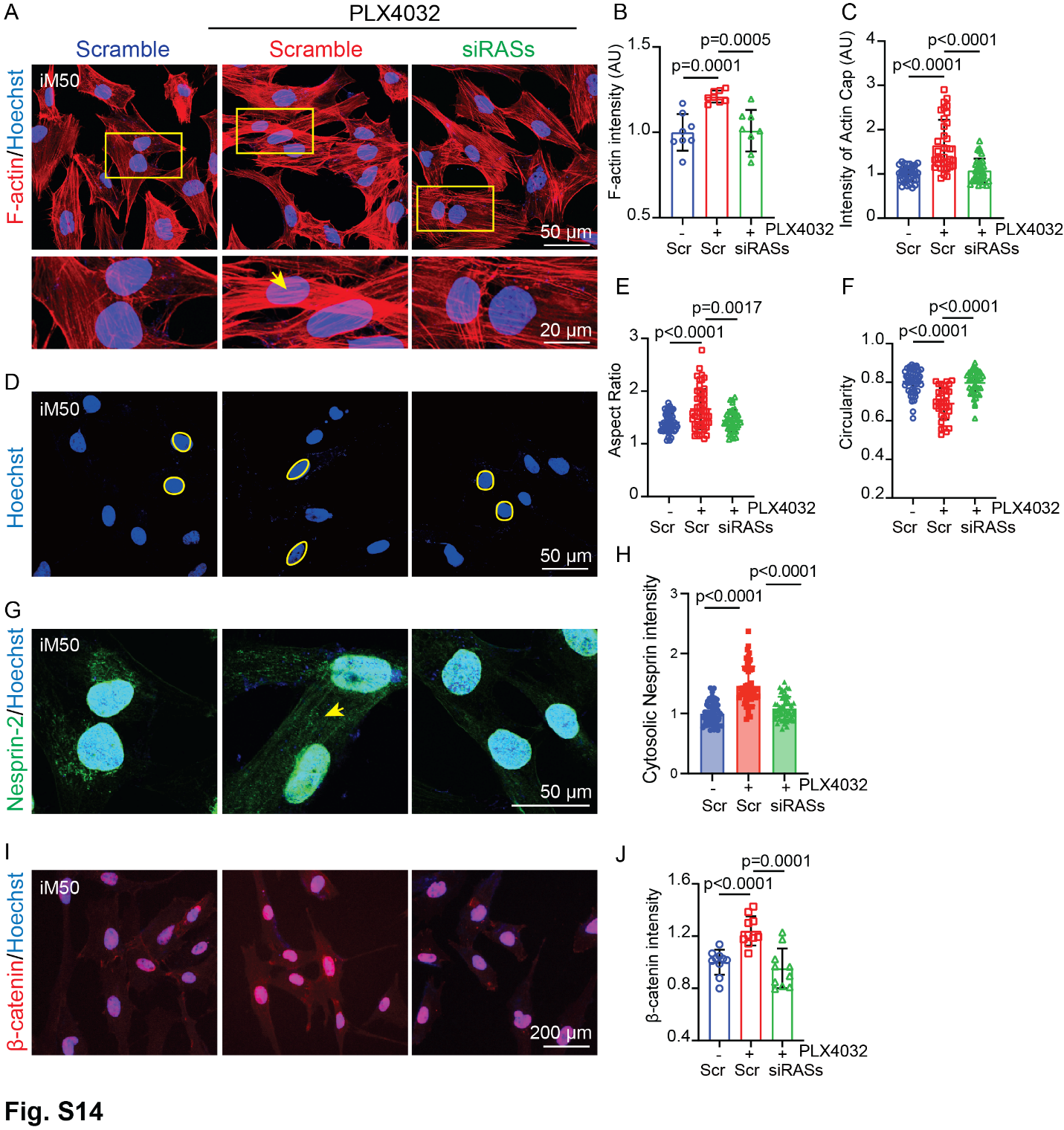
**

**Supplementary Figure S14. BRAFi-induced nuclear deformation and β-catenin accumulation are RAS dependent**

(A) Confocal images showing F-actin expression and organization in iM50 cells transfected with scramble siRNA, scramble iM50 treated with PLX4032 (scramble), and RAS-deficient iM50 (siRASs) treated with PLX4032. Insets display enlarged views of representative individual cells (highlighted by yellow boxes). Yellow arrows indicate actin caps. Scale bars are as indicated.

(B, C) Quantification of F-actin intensity (B) and actin cap intensity (C) using ImageJ (n = 8 randomly selected 20× fields per group for B; n = 34–35 nuclei per group for C).

(D) Representative confocal images of nuclei visualized by Hoechst staining in iM50 cells transfected with scramble siRNA, scramble iM50 treaded with PLX4032, and RAS-deficient iM50 (siRASs) treated with PLX4032. Nuclear boundaries are outlined with yellow circles. Scale bar: 50 μm.

(E, F) Quantification of nuclear morphology based on the confocal images shown in (D), including nuclear aspect ratio (E) and circularity (F), performed using ImageJ. Data are presented as mean ± SD (n = 35–52 nuclei per group).

(G) Confocal images showing Nesprin-2 distribution in iM50 cells transfected with scramble siRNA, scramble iM50 treaded with PLX4032, and RAS-deficient iM50 (siRASs) treated with PLX4032. Yellow arrow indicates abnormal cytosolic localization of Nesprin-2. Scale bar: 50 μm.

(H) Quantification of cytosolic Nesprin-2 intensity. Data are presented as mean ± SD (n = 52–55 CAFs per group)

(I) Representative fluorescence images showing nuclear β-catenin staining in iM50 cells transfected with scramble siRNA, scramble iM50 treaded with PLX4032, and RAS-deficient iM50 (siRASs) treated with PLX4032. Scale bar: 200 μm.

(J) Quantification of nuclear β-catenin intensity under the indicated conditions shown in (I). Data are presented as mean ± SD. n = 10 randomly selected 20×fields per group.
