# Supplementary material for: BRAF Inhibition–Associated Nuclear Remodeling is Linked to Cancer-Associated Fibroblast Activation
Source: Cancer Res Commun. 2026 Jul 16;6(7):1693–713. doi: 10.1158/2767-9764.CRC-25-0682 (PMC13373777; doi:10.1158/2767-9764.CRC-25-0682)
Supplement: Supplementary Figure S15 — Figure S15. ERK signaling is downstream of BRAFi-induced RAF activation [file crc-25-0682_supplementary_figure_s15_suppsf15.docx]

**
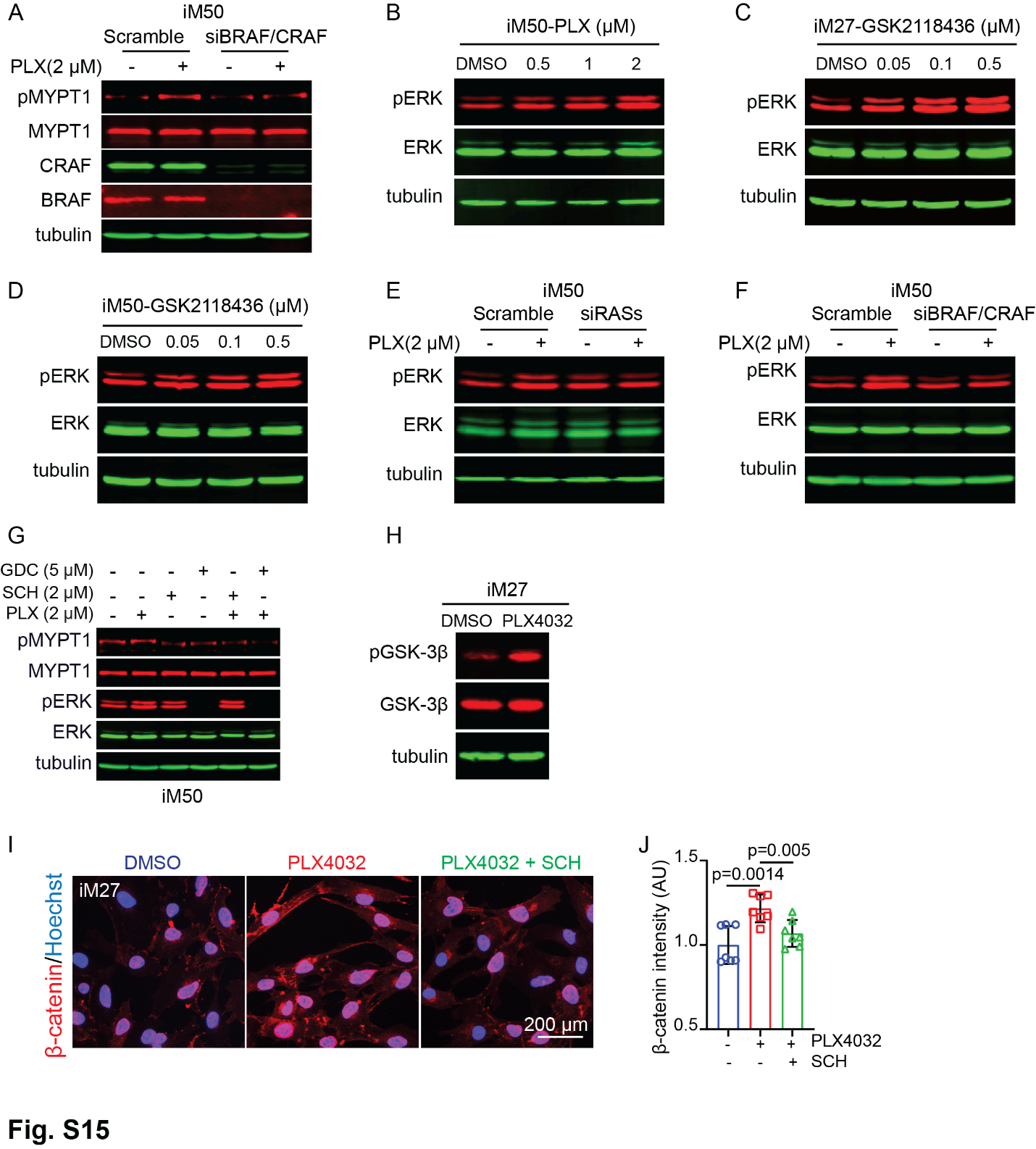
**

**Supplementary Figure S15. ERK signaling is downstream of BRAFi-induced RAF activation**

(A) Western blot showing MYPT1phosphorylation in iM50 cells transfected with scramble siRNA or BRAF/CRAF siRNA (siBRAF/CRAF), with or without PLX4032 treatment.

(B) Western blot showing increased ERK phosphorylation in iM50 cells in response to increasing PLX4032 concentrations compared with DMSO-treated cells.

(C, D) Western blot showing increased ERK phosphorylation in response to increasing concentrations of GSK2118436 in iM27 cells (C) and iM50 cells (D).

(E) Western blot showing ERK phosphorylation in scramble siRNA-transfected iM50 cells and RAS-deficient-iM50 cells (siRASs) with or without PLX4032 treatment.

(F) Western blot showing ERK phosphorylation in iM50 transfected with scramble siRNA and BRAF/CRAF siRNA (siBRAF/CRAF) with or without PLX4032 treatment.

(G) Western blot showing MYPT1phosphorylation in iM50 cells treated with DMSO, PLX4032, ERK inhibitor SCH772984 (SCH), MEK inhibitor GDC0973 (GDC), or a combination of PLX4032 with SCH or GDC.

(H) Western blot showing GSK-3β phosphorylation in iM27 cells with or without PLX4032 treatment.

(I) Fluorescence microscopy images showing nuclear β-catenin expression in iM27 cells treated with DMSO, PLX4032, or a combination of PLX4032 and the ERK inhibitor SCH772984. Scale bar: 200 μm.

(J) Quantification of nuclear β-catenin intensity in iM27 cells corresponding to (I). Data are presented as mean ± SD (n = 7 randomly selected 20× fields per group).
