# Supplementary material for: BRAF Inhibition–Associated Nuclear Remodeling is Linked to Cancer-Associated Fibroblast Activation
Source: Cancer Res Commun. 2026 Jul 16;6(7):1693–713. doi: 10.1158/2767-9764.CRC-25-0682 (PMC13373777; doi:10.1158/2767-9764.CRC-25-0682)
Supplement: Supplementary Figure S16 — Figure S16. ROCK inhibition reverses PLX4032-induced nuclear deformation, actin polymerization, and β-catenin nuclear accumulation in CAFs [file crc-25-0682_supplementary_figure_s16_suppsf16.docx]

**
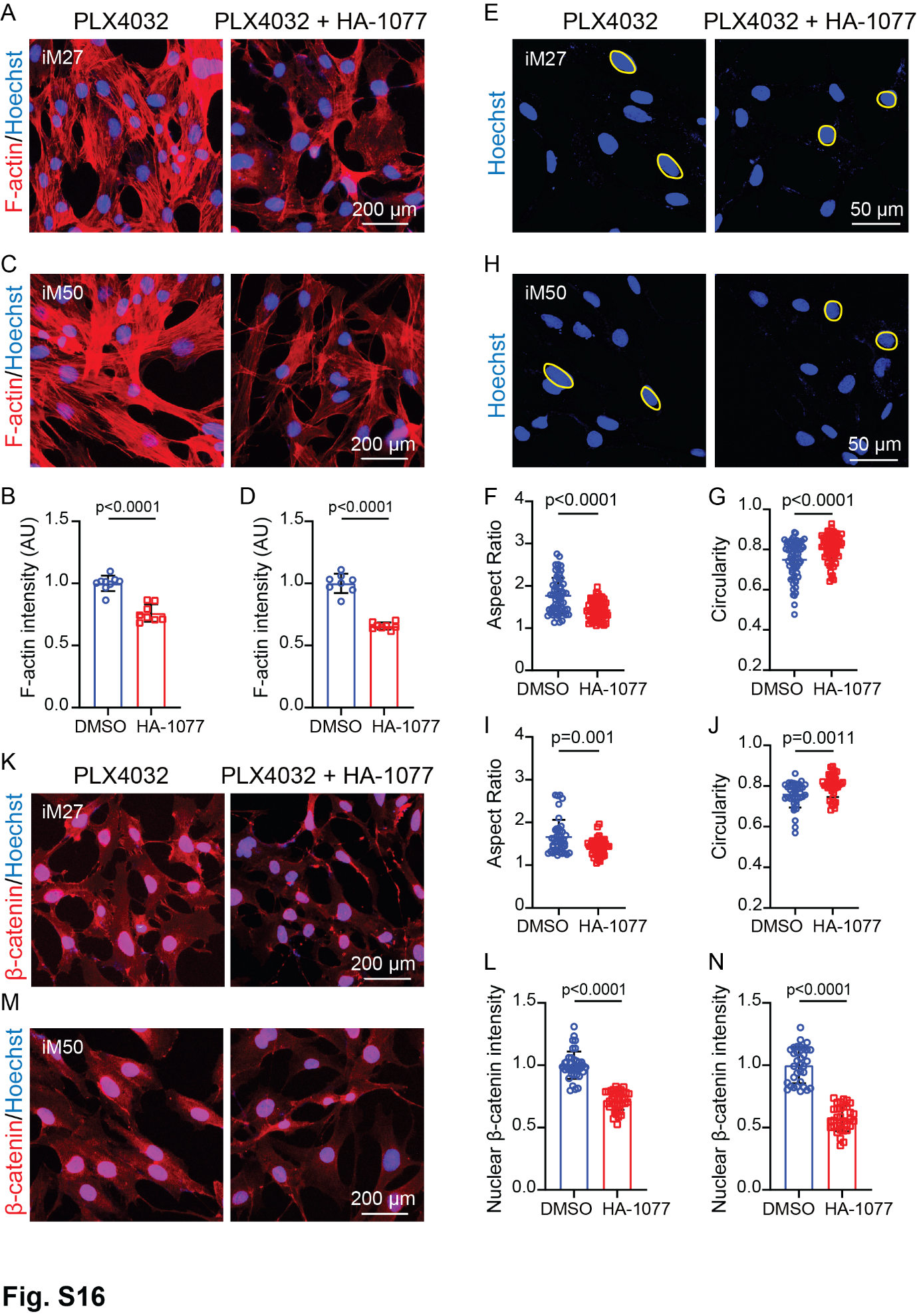
**

**Supplementary Figure S16. ROCK inhibition reverses PLX4032-induced nuclear deformation, actin polymerization, and β-catenin nuclear accumulation in CAFs**

(A, C) Representative fluorescence images of F-actin staining in iM27 cells (A) and iM50 cells (C) treated with PLX4032 or a combination of PLX4032 and HA-1077. Scale bar: 200 μm.

(B, D) Quantification of F-actin intensity in iM27 cells (B) and iM50 cells (D) under the indicated conditions corresponding to (A) and (C), respectively. Data are presented as mean ± SD (n=8–9 randomly selected 20× fields per group).

(E, H) Representative confocal images showing nuclear morphology visualized by Hoechst staining in iM27 cells (E) and in iM50 cells (H) treated with PLX4032 or a combination of PLX4032 and the ROCK inhibitor HA-1077. Scale bar: 50 μm.

(F, G) Quantification of nuclear morphology in iM27 cells, including nuclear aspect ratio (F) and circularity (G), performed using ImageJ based on the corresponding images in (E). Data are presented as mean ± SD (n = 39–64 nuclei per group).

(I, J) Quantification of nuclear morphology in iM50 cells, including nuclear aspect ratio (I) and circularity (J), performed using ImageJ based on the corresponding images in (H). Data are presented as mean ± SD (n = 39–64 nuclei per group).

(K, M) Representative fluorescence images showing nuclear β-catenin intensity in iM27 cells (K) and iM50 cells (M) treated with PLX4032 or a combination of PLX4032 and HA-1077. Scale bar: 200 μm.

(L, N) Quantification of nuclear β-catenin intensity in iM27 cells (L) and iM50 cells (N) under the indicated conditions corresponding to (K) and.(M), respectively. Data are presented as mean ± SD (n = 33–40 nuclei per group).
