# Supplementary material for: BRAF Inhibition–Associated Nuclear Remodeling is Linked to Cancer-Associated Fibroblast Activation
Source: Cancer Res Commun. 2026 Jul 16;6(7):1693–713. doi: 10.1158/2767-9764.CRC-25-0682 (PMC13373777; doi:10.1158/2767-9764.CRC-25-0682)
Supplement: Supplementary Figure S17 — Figure S17. ROCK inhibition reverses stiffness-induced actin remodeling, nuclear deformation, and beta-catenin accumulation in CAFs [file crc-25-0682_supplementary_figure_s17_suppsf17.docx]

**
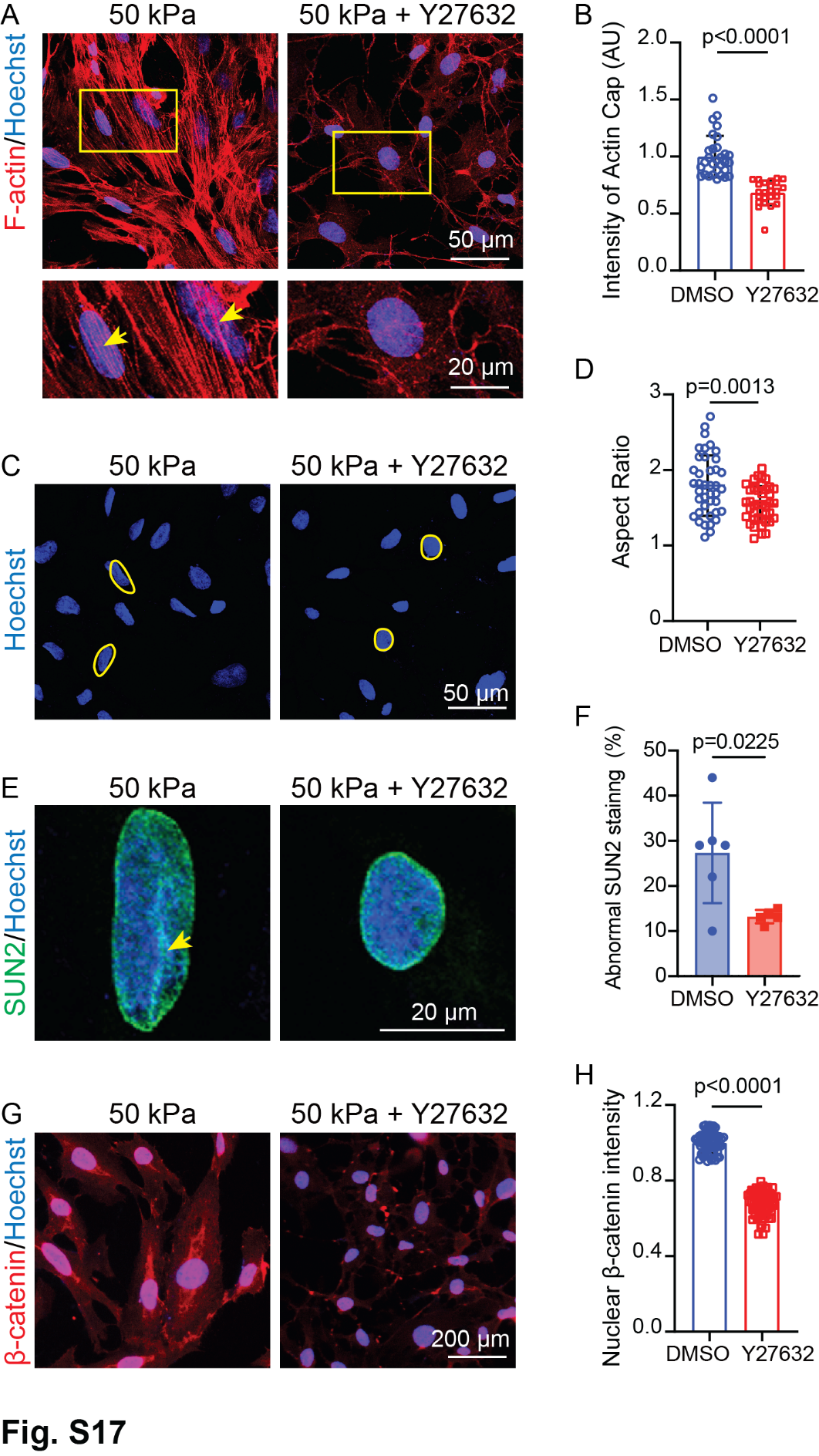
**

**Supplementary Figure S17. ROCK inhibition reverses stiffness-induced actin remodeling, nuclear deformation, and β-catenin accumulation in CAFs**

(A) Confocal images showing F-actin expression and organization in iM27 cells cultured on hard slides with a stiffness of 50 kPa with or without the ROCK inhibitor Y27632. Insets show enlarged views of representative individual cells highlighted by yellow boxes. Yellow arrows indicate actin caps. Scale bars are as indicated.

(B) Quantification of actin cap intensity from (A) using ImageJ. Data are presented as mean ± SD (n = 24–30 nuclei per group).

(C) Representative confocal images of nuclear morphology visualized by Hoechst staining in iM27 cells cultured on hard slides with the stiffness of 50 kPa with or without the ROCK inhibitor Y27632. Yellow circles indicate representative nuclei in each group. Scale bar: 50 μm.

(D) Scatter dot plots showing nuclear morphological changes in iM27 under the indicated conditions. Nuclear aspect ratio was analyzed and quantified from confocal images from (C) using ImageJ. Data are presented as mean ± SD (n = 40 nuclei per group).

(E) Representative confocal images showing SUN2 distribution in iM27 cells cultured on hard slides with the stiffness of 50 kPa with or without the ROCK inhibitor Y27632. Yellow arrow indicates disorganized SUN2 distribution. Scale bar: 20 μm.

(F) Quantification of the percentages of iM27 cells exhibiting abnormal SUN2 staining under indicated conditions. Data are presented as mean ± SD (n = 5–6 random 40× fields).

(G) Representative fluorescence images showing nuclear β-catenin staining in iM27 cells cultured on hard slides with the stiffness of 50 kPa with or without the ROCK inhibitor Y27632. Scale bar: 200 μm.

(H) Quantitative comparison of nuclear β-catenin intensity in iM27 cells cultured under indicated conditions from (G). Data are presented as mean ± SD (n = 60 cells per group).
