# Supplementary material for: BRAF Inhibition–Associated Nuclear Remodeling is Linked to Cancer-Associated Fibroblast Activation
Source: Cancer Res Commun. 2026 Jul 16;6(7):1693–713. doi: 10.1158/2767-9764.CRC-25-0682 (PMC13373777; doi:10.1158/2767-9764.CRC-25-0682)
Supplement: Supplementary Figure S18 — Figure S18. ROCK inhibition reverses stiffness-induced nuclear deformation, actin polymerization, and beta-catenin nuclear accumulation in CAFs [file crc-25-0682_supplementary_figure_s18_suppsf18.docx]

**
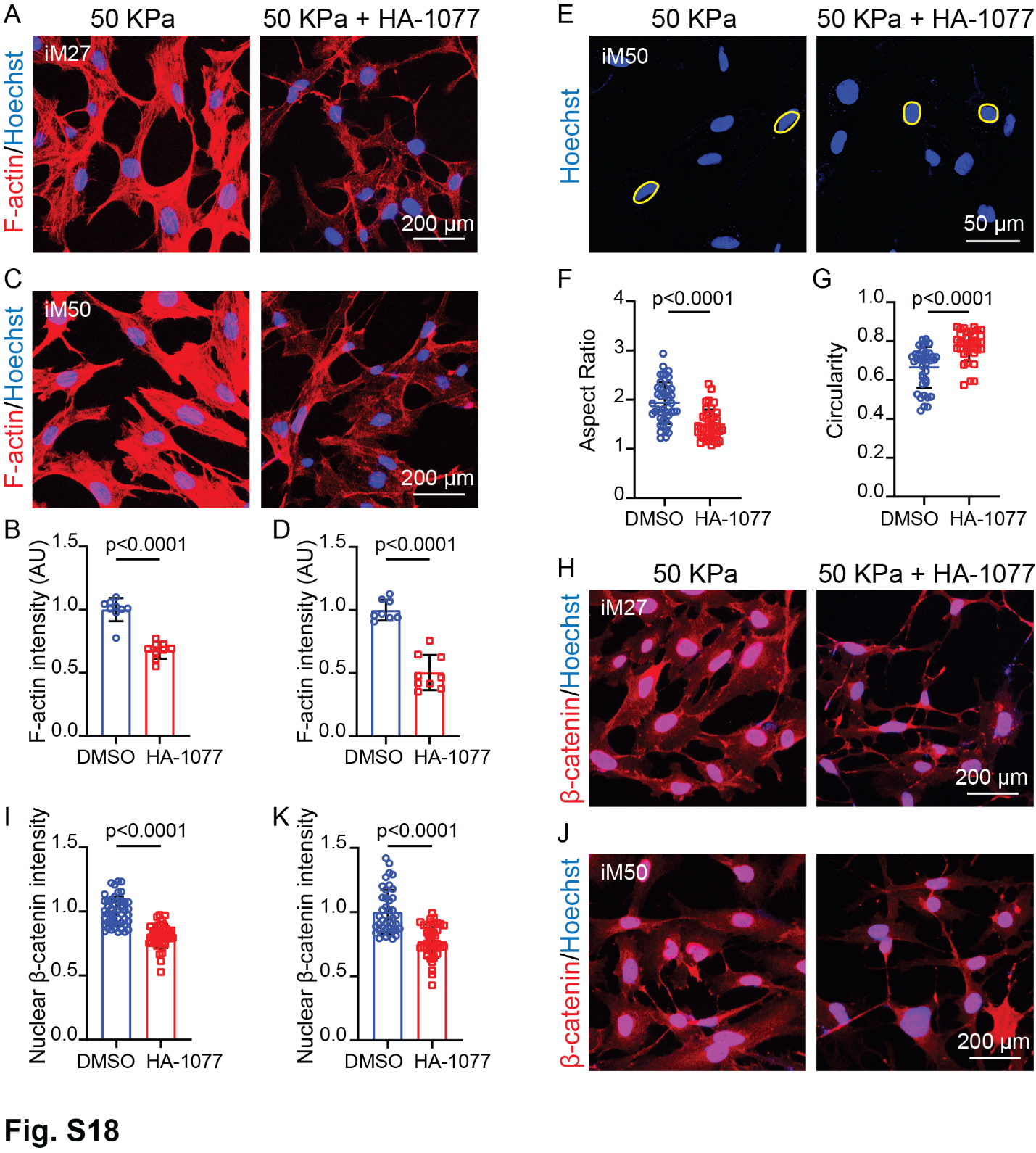
**

**Supplementary Figure S18. ROCK inhibition reverses stiffness-induced nuclear deformation, actin polymerization, and β-catenin nuclear accumulation in CAFs**

(A, C) Fluorescence microscopy images showing F-actin staining in iM27 cells (A) and iM50 cells (C) cultured on hard slides with a stiffness of 50 kPa with or without HA-1077 treatment. Scale bar: 200 μm.

(B, D) Quantification of F-actin intensity in iM27 cells (B) and iM50 cells (D) corresponding to (A) and (C), respectively. Data are presented as mean ± SD (n = 8–9 randomly selected 20× fields per group).

(E) Representative confocal images of Hoechst-stained nuclei in iM50 cells cultured on hard slides with a stiffness of 50 kPa with or without ROCK inhibitor HA-1077 treatment. Yellow circles highlight the approximate nuclear boundaries. Scale bar: 50 μm

(F, G) Scatter dot plots showing the nuclear morphological changes under the indicated conditions corresponding to (E). Nuclear aspect ratio (F) and circularity (G) were analyzed and quantified using ImageJ. Data are presented as mean ± SD (n = 35–43 nuclei per group).

(H, J) Fluorescence microscopy images showing nuclear β-catenin expression in iM27 cells (H) and iM50 cells (J) cultured on hard slides with a stiffness of 50 kPa, with or without HA-1077 treatment. Scale bar: 200 μm.

(I, K) Quantification of nuclear β-catenin intensity in iM27 cells (I) and iM50 cells (K) corresponding to (H) and (J), respectively. Data are presented as mean ± SD (n = 40–48 cells per group).
