# Supplementary material for: BRAF Inhibition–Associated Nuclear Remodeling is Linked to Cancer-Associated Fibroblast Activation
Source: Cancer Res Commun. 2026 Jul 16;6(7):1693–713. doi: 10.1158/2767-9764.CRC-25-0682 (PMC13373777; doi:10.1158/2767-9764.CRC-25-0682)
Supplement: Supplementary Figure S19 — Figure S19. BRAFi promotes BRAF and CRAF dimerization and transactivation in CAFs [file crc-25-0682_supplementary_figure_s19_suppsf19.docx]

**Supplementary Figure S19. BRAFi promotes BRAF and CRAF dimerization and transactivation in CAFs**

(Left) In BRAF wild-type cells without BRAFi treatment, extracellular signals transmitted through receptor tyrosine kinases (RTKs) activate the small GTPase RAS. Activated RAS recruits BRAF and CRAF to the plasma membrane, relieving autoinhibition and promoting RAF dimerization and transactivation, which subsequently activates downstream MEK-ERK signaling.

(Middle) In BRAF-mutant cells, mutant BRAF contains a constitutively active kinase domain that activates downstream MEK and ERK independently of RAS activation. BRAFi are designed to bind the mutant kinase domain and inhibit its catalytic activity.

(Right) In BRAF wild-type CAFs, BRAF and CRAF require RAS binding to relieve autoinhibition because their kinase domains lack the V600E mutation. When BRAFi bind to the kinase domains of BRAF or CRAF, they induce conformational changes that promote BRAF–CRAF dimerization and transactivation, resulting in enhanced downstream MEK-ERK signaling. Created in BioRender. Siegfried, L. (2026) https://BioRender.com/ie7p9ak.
